# Kinase-Associated Phosphoisoform Assay: a novel candidate-based method to detect specific kinase-substrate phosphorylation interactions in vivo

**DOI:** 10.1186/s12870-016-0894-1

**Published:** 2016-09-21

**Authors:** Magdalena Dory, Zoltán Doleschall, Szilvia K. Nagy, Helga Ambrus, Tamás Mészáros, Beáta Barnabás, Róbert Dóczi

**Affiliations:** 1 Department of Plant Cell Biology, Centre for Agricultural Research of the Hungarian Academy of Sciences, H-2462, Brunszvik u. 2, Martonvásár, Hungary; 2 Department of Pathogenetics, National Institute of Oncology, H-1122, Ráth György u. 7-9, Budapest, Hungary; 3 Department of Medical Chemistry, Molecular Biology and Pathobiochemistry, Semmelweis University, H-1094, Tűzoltó u. 37-47, Budapest, Hungary; 4 Research Group for Technical Analytical Chemistry, Hungarian Academy of Sciences - Budapest University of Technology and Economics, H-1111, Szt. Gellért tér 4, Budapest, Hungary

**Keywords:** Protein kinase, Phosphorylation assay, Signal transduction, Protoplast transfection, Capillary isoelectric focusing, Nanofluidic immunoassay, APETALA 2, WUSCHEL, Arabidopsis thaliana

## Abstract

**Background:**

Protein kinases are important components of signalling pathways, and kinomes have remarkably expanded in plants. Yet, our knowledge of kinase substrates in plants is scarce, partly because tools to analyse protein phosphorylation dynamically are limited. Here we describe Kinase-Associated Phosphoisoform Assay, a flexible experimental method for directed experiments to study specific kinase-substrate interactions in vivo.

The concept is based on the differential phosphoisoform distribution of candidate substrates transiently expressed with or without co-expression of activated kinases. Phosphorylation status of epitope-tagged proteins is subsequently detected by high-resolution capillary isoelectric focusing coupled with nanofluidic immunoassay*,* which is capable of detecting subtle changes in isoform distribution.

**Results:**

The concept is validated by showing phosphorylation of the known mitogen-activated protein kinase (MAPK) substrate, ACS6, by MPK6. Next, we demonstrate that two transcription factors, WUS and AP2, both of which are shown to be master regulators of plant development by extensive genetic studies, exist in multiple isoforms in plant cells and are phosphorylated by activated MAPKs.

**Conclusion:**

As plant development flexibly responds to environmental conditions, phosphorylation of developmental regulators by environmentally-activated kinases may participate in linking external cues to developmental regulation. As a counterpart of advances in unbiased screening methods to identify potential protein kinase substrates, such as phosphoproteomics and computational predictions, our results expand the candidate-based experimental toolkit for kinase research and provide an alternative in vivo approach to existing in vitro methodologies.

**Electronic supplementary material:**

The online version of this article (doi:10.1186/s12870-016-0894-1) contains supplementary material, which is available to authorized users.

## Background

During evolution, phosphorylation emerged as a prominent type of post-translational modification, because of its versatility and ready reversibility [1]. Due to sessile lifestyle, kinomes have remarkably expanded in the plant kingdom: in *Arabidopsis* and rice four percent of genes encode kinases [2, 3], whereas in the human genome this number is 2 % [4]. Although kinases are overrepresented in plants, and despite their obvious importance in key processes, knowledge on actual plant protein kinase substrates is seriously lagging behind those of animal kinases. Mitogen-activated protein kinases (MAPKs) are very good examples: plant MAPKs are most similar to human ERK-type MAPKs, and while well over 150 ERK1/2 substrates are known [5], there are only about twenty individually characterised substrates described in the model plant *Arabidopsis* [6, 7]. Due to independent evolution of MAPK signalling networks in different kingdoms, homology-based substrate search is not a suitable option, known plant MAPK substrates have been identified using specific and targeted techniques, such as yeast two-hybrid screens. Therefore the generation of novel tools for analysing cellular protein phosphorylation is critical in order to efficiently dissect plant kinase networks [8].

Technical advances in kinase research have primarily focused on phosphoproteomics related technologies [9] and thus have resulted in various screening methods. However, genes expressed at low levels or in rare cell types are easily missed by such methods. Advances in bioinformatics and systems biology can deliver solutions to this issue by efficient prediction of underrepresented substrates. Accordingly, in vitro MAPK substrates were reported using protein microarrays [10, 11] and phosphoproteomics [12], and a consensus phosphorylation sequence for MPK3 and MPK6 was identified by screening a random positional peptide library, which was consequently used for predicting novel candidate MAPK substrates [13] in *Arabidopsis*.

Nevertheless, whether identified by in vivo or in silico screening, at least a subset of the substrate proteins has to be verified by targeted experiments. Yet, development of unbiased discovery tools has not been followed by a corresponding improvement of candidate-based approaches. Protein phosphorylation is commonly demonstrated by in vitro kinase assay, a method developed decades ago, without substantive improvement since. This is a tedious method, involving protein affinity purification, and entails use of hazardous radioisotopes. Moreover, the use of high concentrations of purified kinases and the lack of cellular regulatory mechanisms in vitro relatively often lead to false results [9, 14]. Therefore it is timely to develop alternative methods capable of addressing in vivo phosphorylation interactions in a targeted manner.

Capillary isoelectric focusing (cIEF) coupled nanofluidic immunoassay has been developed to detect differentially present protein isoforms in cellular protein samples [15]. In this system, protein isoforms of varying isoelectric points are separated by isoelectric focusing in a capillary, immobilised by UV light, and immunoprobed with antibodies. Chemiluminescent signal generated by antibody-coupled enzyme is captured by a sensitive CCD camera. However, scarcity of specific antibodies means a serious bottleneck for the application of immunodetection-based assays in plants.

Combination of cIEF-immunoassay with transient expression of fusion-protein constructs in protoplasts offers two important advantages: i) it circumvents the issue of limited availability of specific antibodies, ii) transfection enables the co-expression of investigated proteins with active or inactive protein kinases to study specific kinase-substrate relationships of interest. Protoplasts are commonly used to demonstrate protein-protein interactions, and have also been applied to study MAPK-substrate interactions, e.g. [16–19]. Here we demonstrate that detection of changes in phosphoisoform distribution of transfected fusion proteins by cIEF-immunoassay is a suitable approach to study in vivo kinase-substrate phosphorylation interactions in plants, by showing phosphorylation of a known and two novel MAPK substrates.

## Results

### Phosphorylation of the known MAPK substrate, ACS6, is detected by the novel method

**Fig. 1:**
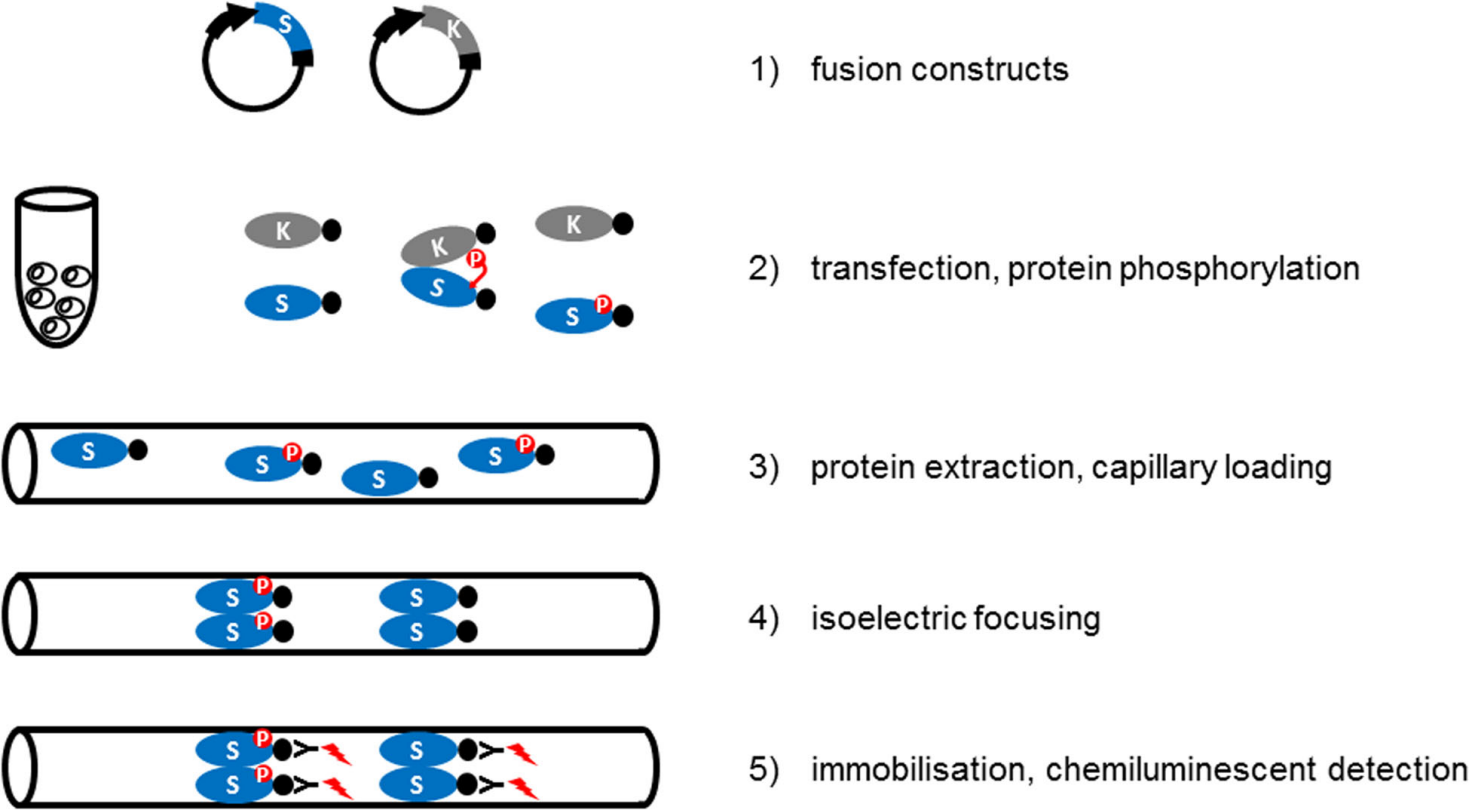
Experimental setup of Kinase-Associated Phosphoisoform Assay. The concept is based on the differential phosphoisoform distribution of candidate substrates transiently expressed with or without co-expression of activated kinases. Full-length cDNAs of protein kinase(s) and candidate substrate(s) are cloned into plant expression vectors as translational fusion constructs. Use of fusion proteins containing commonly used epitopes also circumvents the need of specific antibodies. Candidate substrates are transfected into protoplasts, where intracellular phosphorylation can occur. Following an appropriate incubation period the protoplasts are harvested, lysed and the protein extracts are loaded into capillaries. Isoelectric focusing takes place in a pH gradient within the capillaries. Finally, separated proteins are immobilised to the capillary surface, and detected by chemiluminescent enzyme-coupled antibodies, specific against the epitopes fused to the substrate proteins

**Fig. 2:**
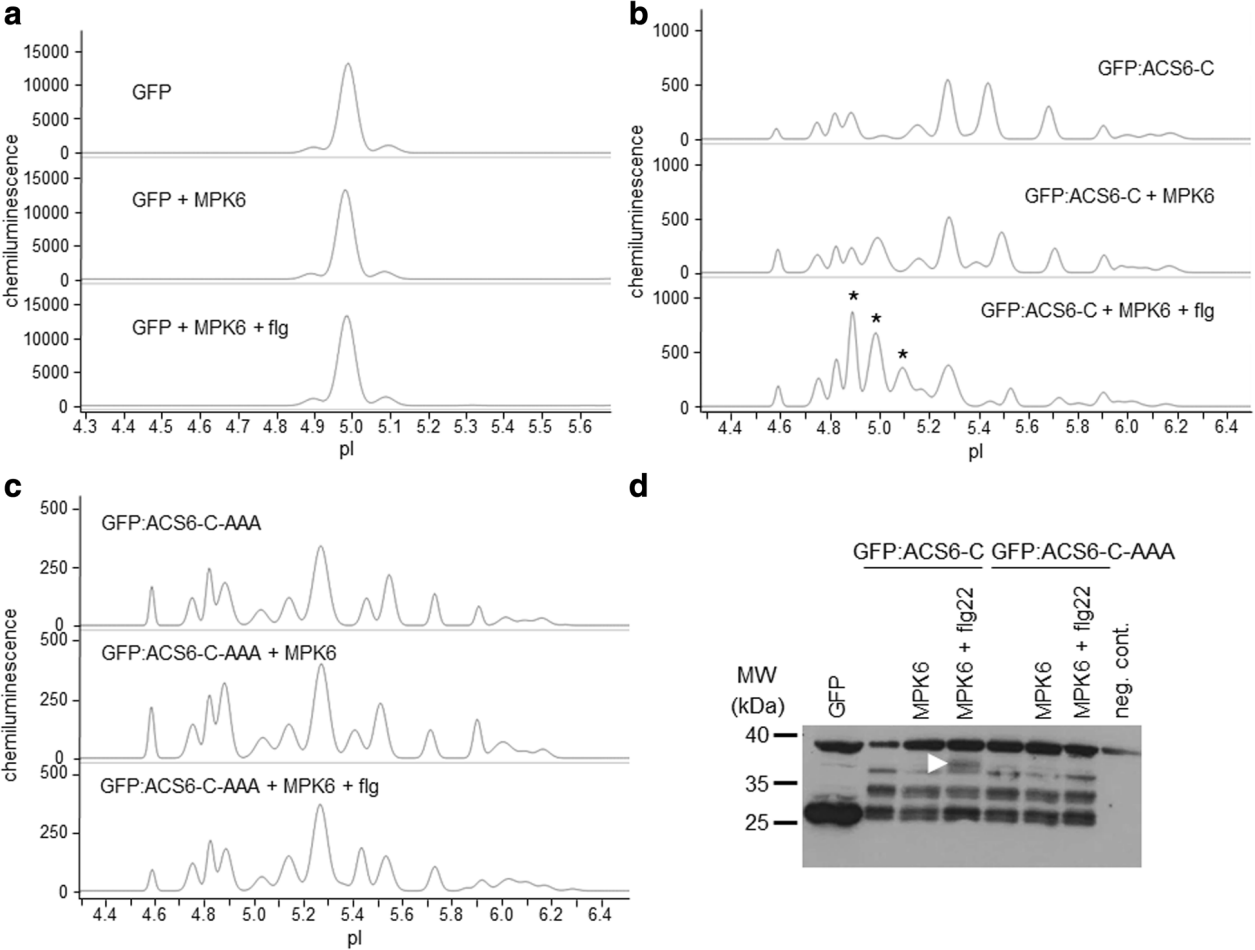
Detection of phosphoisoform distribution of transiently expressed GFP variants by cIEF-immunoassay. **a**-**c** Electropherograms of various GFP-fusion proteins and their isoform distributions in cIEF-immunoassay. Expressed proteins and treatments are indicated for each sample. **a** Unmodified GFP is present in one major isoform and is not phosphorylated by MPK6. Top: control (single GFP construct transformation), middle: GFP co-expressed with MPK6, bottom: GFP co-expressed with flg22-activated MPK6. **b** Isoform distribution of GFP:ACS-C (the C-terminal domain of ACS6 fused to the C terminus of GFP). Top: control (single GFP:ACS-C construct transformation), middle: GFP:ACS-C co-expressed with MPK6, bottom: GFP:ACS-C co-expressed with flg22-activated MPK6. Asterisks indicate acidic isoforms specifically accumulating in the presence of activated MPK6. **c** Isoform distribution of a GFP:ACS-C variant, which is nonphosphorylatable by MAPKs (GFP:ACS-C-AAA). Top: control (single GFP:ACS-C-AAA construct transformation), middle: GFP:ACS-C-AAA co-expressed with MPK6, bottom: GFP:ACS-C-AAA co-expressed with flg22-activated MPK6. **d** Conventional SDS-PAGE immunoblot of transiently expressed GFP variants. Arrowhead indicates a band, which specifically accumulates in the presence of activated MPK6. Negative control (neg. cont.) denotes a protoplast sample not transfected with GFP

In order to provide a cellular alternative to the often unreliable in vitro kinase assay to study specific kinase-substrate interactions, we have optimised a cIEF-coupled nanofluidic immunoassay to detect differentially present protein isoforms in transfected protoplasts with or without co-expression of active protein kinases and designated the concept Kinase-Associated Phosphoisoform Assay (Fig. 1). For primary testing of the concept we first assayed phosphorylation of the C-terminal domain of 1-AMINOCYCLOPROPANE-1-CARBOXYLIC ACID (ACC) SYNTHASE 6, ACS6 (ACS-C), a known substrate of the MAP kinase MPK6 [20]. Protoplasts were transformed either with green fluorescent protein (GFP) or a construct consisting of ACS-C fused to the C-terminus of GFP (GFP:ACS-C). GFP is predominantly present in a single isoform (Fig. 2a), whereas GFP:ACS-C is detected as several peaks of different isoelectric point (pI) values implying the parallel presence of differentially phosphorylated isoforms (Fig. 2b, Table 1). Although, in silico analysis can predict phosphorylation with limited reliability, various putative phosphorylation sites in the C-terminus of ACS6 are identified by the Eukaryotic Linear Motif (ELM) Resource [21], (Additional file 1: Table S1), indicating intense and dynamic phosphorylation, in good agreement with our observations. The complex isoform distribution could be reduced by phosphatase treatment of the protein extracts (Additional file 2: Fig. S1). Co-expression of MPK6 in protoplasts treated with the bacterial flagellin-derived elicitor peptide flg22 [22], an activator of MPK6, resulted in the marked accumulation of acidic isoforms, most significantly the isoforms of pI 4.9, 5.0 and 5.1 (Fig. 2b, Table 1), indicating de novo protein phosphorylation. Co-expression with non-activated MPK6 also brought about acidification to a lesser extent, which primarily manifested in the accumulation of the pI 5.0 isoform. As a negative control, a mutant GFP:ACS-C variant deprived of the MAPK phosphorylation sites (S46A, S49A, S54A) was also co-expressed with MPK6, but its isoform distribution was unaffected by MPK6 (Fig. 2c, Table 1). Similarly, neither unfused GFP is phosphorylated by MPK6 (Fig. 2a). In comparison, transfected proteins were also detected by conventional SDS-PAGE immunoblot, where a slower-migrating band appeared in the GFP:ACS-C sample co-transformed with activated MPK6 (Fig. 2d). Thus, using a known MAPK substrate we have demonstrated that it is possible to detect protein (hyper)phosphorylation by a co-expressed active kinase by transfection-coupled cIEF-immunoassay, even if the protein exists in multiple phosphorylated isoforms in the cellular context.

**Table 1:** Peak data for ACS6 C-terminal domain phosphorylation

pI	Area %: GFP:ACS6-C +			pI	Area %: GFP:ACS6-C-AAA +		
	-	MPK6	MPK6 + flg		-	MPK6	MPK6 + flg
4.58	2.7	5.46	3.09	4.59	5.13	6.05	3.09
4.75	6.04	7.23	7.19	4.75	7	7.89	7.21
4.82	9.64	7.33	10.02	4.82	11.02	9.04	9.1
4.89	13.35	9.43	21.45	4.88	16.48	18.74	13.1
5.01	1.54	17.04	20.56	5.03	4.85	5.25	4.48
5.11	0	0	11.44	5.15	8.04	7.93	10.2
5.15	7.09	6.32	4.29				
5.28	23.14	20.46	14.36	5.27	25.15	23.1	27.82
5.39	0	4.73	0	5.39	0	6.4	0
5.44	23.08	14.98	1.58	5.45	5.8	0	8.66
5.55	0	0	3.67	5.55	11.24	10.93	10.48
5.68	11.53	7.02	0				
5.74	0	0	2.34	5.73	5.28	4.67	5.86

Data correspond to the electropherograms shown in Fig. 2b, c

### WUS is an MPK3 substrate in vivo

Initial advances in plant MAPK research predominantly revealed their functions in stress responses, yet, the essential roles of MAPK signalling in plant development are increasingly evident [8]. As most of the identified substrates are also defence related, we aimed at identifying novel substrates with developmental function. We took advantage of the conservation of MAPK docking sites [23], and screened key developmental regulator transcription factors for the presence of the D-site motif as an indicator of possible MAPK interaction [24].

**Fig. 3:**
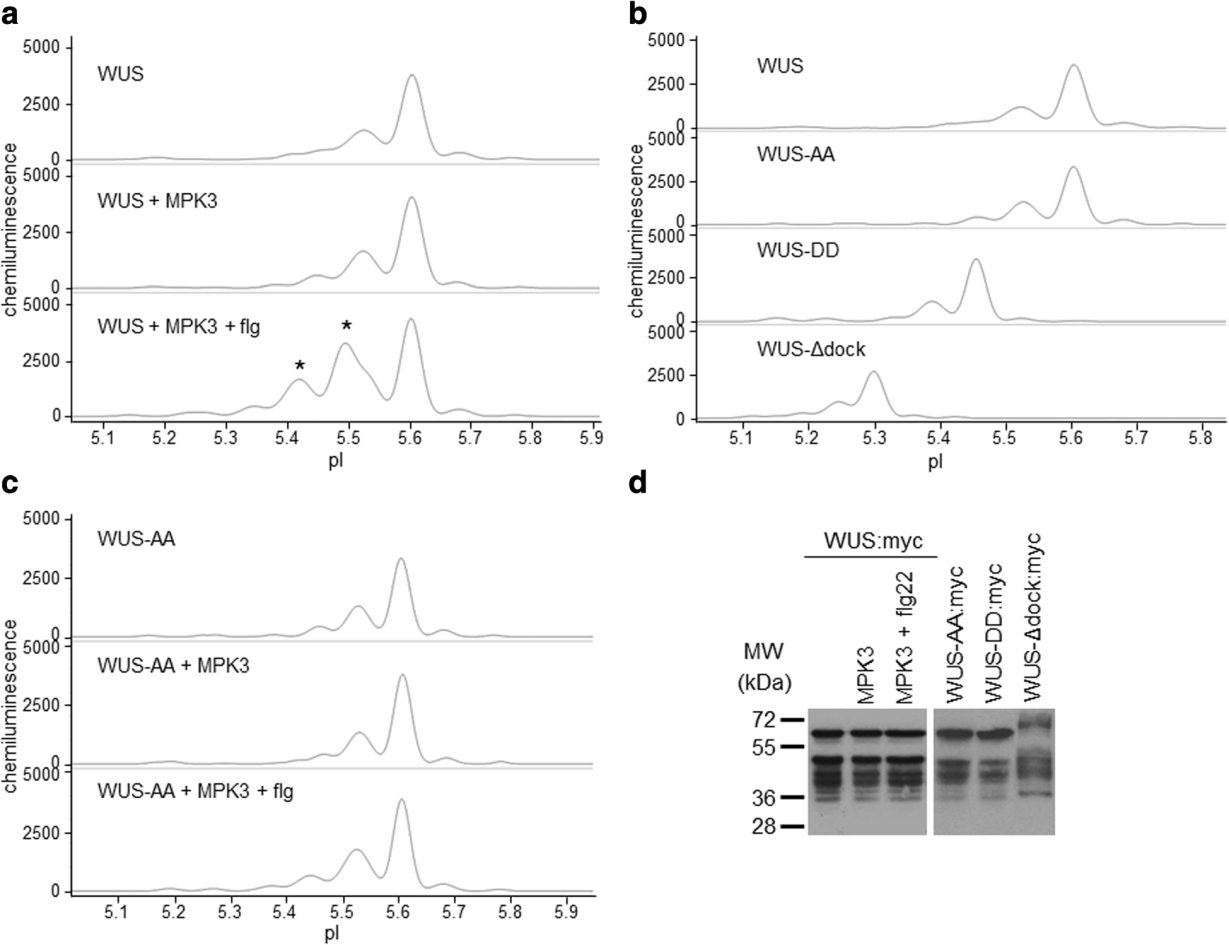
WUS is an MPK3 substrate in vivo. **a**-**c** Electropherograms of various WUS:myc fusion proteins and their isoform distributions in cIEF-immunoassay. Expressed proteins and treatments are indicated for each sample. **a** Effect of activated MPK3 on C-terminal myc-tagged WUS isoform distributions in cIEF-immunoassay. Top: control (single WUS:myc construct transformation), middle: WUS:myc co-expressed with MPK3, bottom: WUS:myc co-expressed with flg22-activated MPK3. Asterisks indicate acidic isoforms specifically accumulating in the presence of activated MPK3. **b** Differential charge compositions of point mutant WUS variants are detected as changes in protein pI values. WUSAA: non-phosphorylatable mutant, WUS-DD: phosphomimetic mutant, WUS-Δdock: MAPK docking D-site disabled mutant. **c** Alanine substitutions at the MAPK phosphorylation sites T108, S112 impair WUS phosphorylation by MPK3. **d** Conventional SDS-PAGE immunoblot of transiently expressed WUS variants

WUS is a key transcription factor controlling the stem cell pool in shoot and floral meristems [25, 26]. This factor is characterised in great detail by genetic methods, yet nothing is known about post-translational modifications of WUS. WUS contains an RRTLPL motif, which may serve as a MAPK docking D-site, and four potential MAPK phosphorylation sites (Additional file 3: Table S2). Here we show that WUS exists in two major isoforms using both GFP and myc epitope tagged WUS constructs (Fig. 3a, Table 2 and Additional file 4: Fig. S2a). Next we tested whether co-expression with active MAPKs results in WUS phosphorylation. To this end WUS was co-expressed with four MAPKs, representing three phylogenetic groups of plant MAPKs (Fig. 3a, Table 2 and Additional file 4: Fig. S2a-c). The marked accumulation of more acidic WUS isoforms indicates that WUS is specifically phosphorylated by MPK3, but not by the related MPK6 of group A, nor by MPK11 (group B) and MPK1 (group C). The MPK3-triggered phosphorylation event could not be brought about by flg22 treatment without MPK3 co-expression, nor by co-expression with non-activated MPK3. As an additional negative control, an inactive MPK3 variant was used in flg22-treated samples, without affecting any isoform redistribution (Additional file 4: Fig. S2c). These results strongly imply that WUS is an MPK3 substrate in vivo.

**Table 2:** Peak data for WUS phosphorylation

pI	Area %: WUS +			pI	Area %: WUS-AA +		
	-	MPK3	MPK3 + flg		-	MPK3	MPK3 + flg
5.19	1.05	0.74	1.14	5.19	2.21	2	1.44
5.26	0.02	0.33	1.8	5.27	1.48	0.79	1.4
5.36	0	1.85	4.03	5.38	1.7	0.44	3.21
5.41	5.76	9.14	18.3				
5.49	0	0	24.17	5.46	8.67	6.93	11.52
5.52	31.31	30.63	15.42	5.53	28.07	27.88	33.33
5.61	54.84	52.13	31.53	5.61	51.61	56.59	43.73
5.68	5.98	4.46	3.1	5.68	5.28	4.18	4.47
5.77	1.04	0.7	0.51	5.77	0.98	1.19	0.89
pI	Area %: WUS-DD +			pI	Area %: WUS-Δdock +		
	-	MPK3	MPK3 + flg		-	MPK3	MPK3 + flg
5.15	7.39	2.26	3.21	4.95	1.04	2.55	2.11
5.23	6.71	3.65	4.71	5.12	3.76	3.35	5.9
5.32	11.19	11.56	15.47	5.19	7.5	7.84	15.88
5.39	29.76	36.32	36.44	5.25	24.48	28.34	33.19
5.45	43.21	42.89	36.92	5.30	57.25	53.18	38.93
5.53	1.49	2.34	2.42	5.36	4.15	3.47	3.29
5.61	0.26	0.98	0.84	5.42	1.81	1.26	0.7

Corresponding visualised peak areas used for calculating area percentages are shown in Additional file 5: Fig. S3

To further verify WUS phosphorylation by MPK3, three different mutations affecting MAPK phosphorylation were introduced. The phosphoacceptor residues in two S/TP sites (T108, S112), which lay outside of the homeodomain were substituted either by alanines (WUS-AA, non-phosphorylatable mutant) or by glutamic acids (WUS-DD, phosphomimetic mutant). In a third mutant the putative D-site was disabled (R252E, R253E, L257E: WUS-Δdock). Two of these mutations also altered the pI values calculated by the ExPASy Server [27]. While the WUS-AA mutant has the same theoretical pI as the wild-type protein, the introduced or swapped charges decrease the pI values of WUS-DD and WUS-Δdock. The major peaks of all mutant forms were detected at the expected pI values, demonstrating that subtle differences in protein charge composition are reliably detected (Fig. 3b and Additional files 4 and 5: Figures S2b, S3). The MPK3-mediated acidification of WUS was completely abolished in both the non-phosphorylatable and the phosphomimetic mutants (Fig. 3c, Table 2 and Additional files 4 and 5: Figures S2d and S3b, c). Moreover, phosphorylation was significantly impaired without a functional D-site (Table 2 and Additional files 4 and 5: Figures S2e and S3g), implying direct interaction through this motif. Transfected proteins were also detected by conventional SDS-PAGE immunoblot, which confirmed expression but failed to resolve variations in phosphoisoform distribution (Fig. 3d and Additional file 6: Fig. S4a, b).

**Fig. 4:**
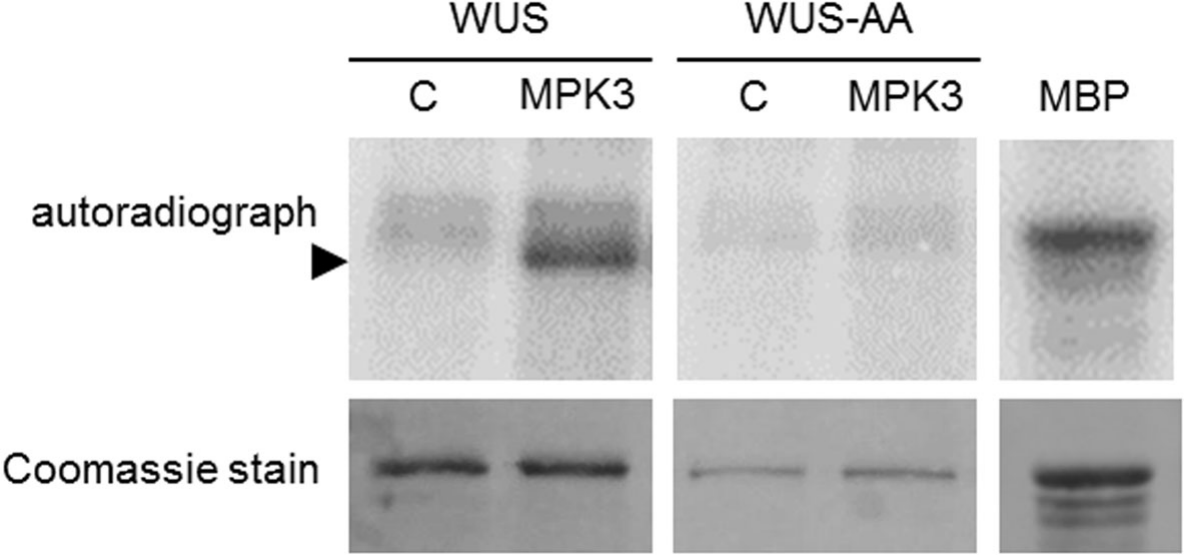
WUS is an MPK3 substrate in vitro. Kinase assay with in vitro translated, affinity purified wild-type GST-WUS (WUS) and T108A, S112A mutant GST-WUS (WUS-AA) variants. C: control, MPK3: WUS variants incubated with in vitro translated, affinity purified, eluted MKK4/MPK3. SDS-PAGE separated WUS protein on the autoradiograph is indicated by arrowhead. Phosphorylation of myelin basic protein (MBP) by MPK3 is shown in the right panel

For comparison, phosphorylation of WUS by MPK3 was also tested by the traditional in vitro kinase assay. In agreement with the above findings, wild-type WUS but not WUS-AA was phosphorylated by MPK3, as indicated by radiolabelled phosphate incorporation (Fig. 4). Conservation of the identified tandem phosphorylation sites in WUS orthologues is shown in Additional file 7: Fig. S5.

### WUS isoforms are consistently detected with various antibodies

In order to make sure that our results are not an artefact of protein tagging or antibody-mediated detection, WUS variants were detected by various antibodies. Although GFP contains one S/TP site, this is located within the globular structure and is most likely inaccessible. In contrast, there are no S/TP sites in the myc tag sequence. Similar WUS isoform distributions were detected with both fusion variants, although in case of the smaller myc tag additional minor peaks could be resolved, therefore this version was studied in more detail (Fig. 3 and Additional file 4: Fig. S2). Phosphatase treatment of the protein extracts resulted in the accumulation of a single WUS isoform (Additional file 2: Fig. S1), confirming protein phosphorylation. To further verify myc-epitope based detection, WUS:myc was detected by three different antibodies with consistent isoform distributions. Routinely, a monoclonal anti-myc antibody directly fused to horseradish peroxidase (Roche) was used, which did not require the use of a secondary antibody. As a consistency control an anti-myc antibody from an independent source (Sigma) was also tested, with identical results (Additional file 6: Fig. S4e). Moreover, a specific anti-WUS antibody is available from Agrisera, which facilitated the choice of WUS as a candidate substrate for testing the fusion-protein-based experimental concept. Indeed, the same isoforms were detected by the specific anti-WUS antibody as with the anti-myc antibodies, both in cIEF-immunoassay and in SDS-PAGE immunoblot (Additional file 6: Fig. S4b and e). These results confirm that tagging and immunodetection do not interfere with intracellular WUS phosphorylation and detection.

Interestingly, WUS fusion variants migrate anomalously in SDS-PAGE. Molecular weights of WUS:myc and WUS:GFP are 48.3 kDa and 60.3 kDa, respectively, however WUS:myc has an apparent molecular weight of about 68 kDa, whereas WUS:GFP migrates at about 54 kDa. Nevertheless, SDS-PAGE migration of differentially phosphorylated isoforms is identical. Similarly, WUS-AA and WUS-DD mutants migrate as their wild-type counterparts, whereas WUS-Δdock migrates slightly slower. Conventional SDS-PAGE is thus not capable to resolve subtle changes in WUS charge composition. Moreover, several faster migrating bands can also be observed, which can be significantly abolished by treating protoplasts with the proteasome inhibitor MG-115, implying that the lower molecular weight bands are degradation products (Additional file 6: Fig. S4c).

The antibodies used in this study are presented in detail in Additional file 8: Table S3.

### AP2 is an MPK6 substrate in vivo

**Fig. 5:**
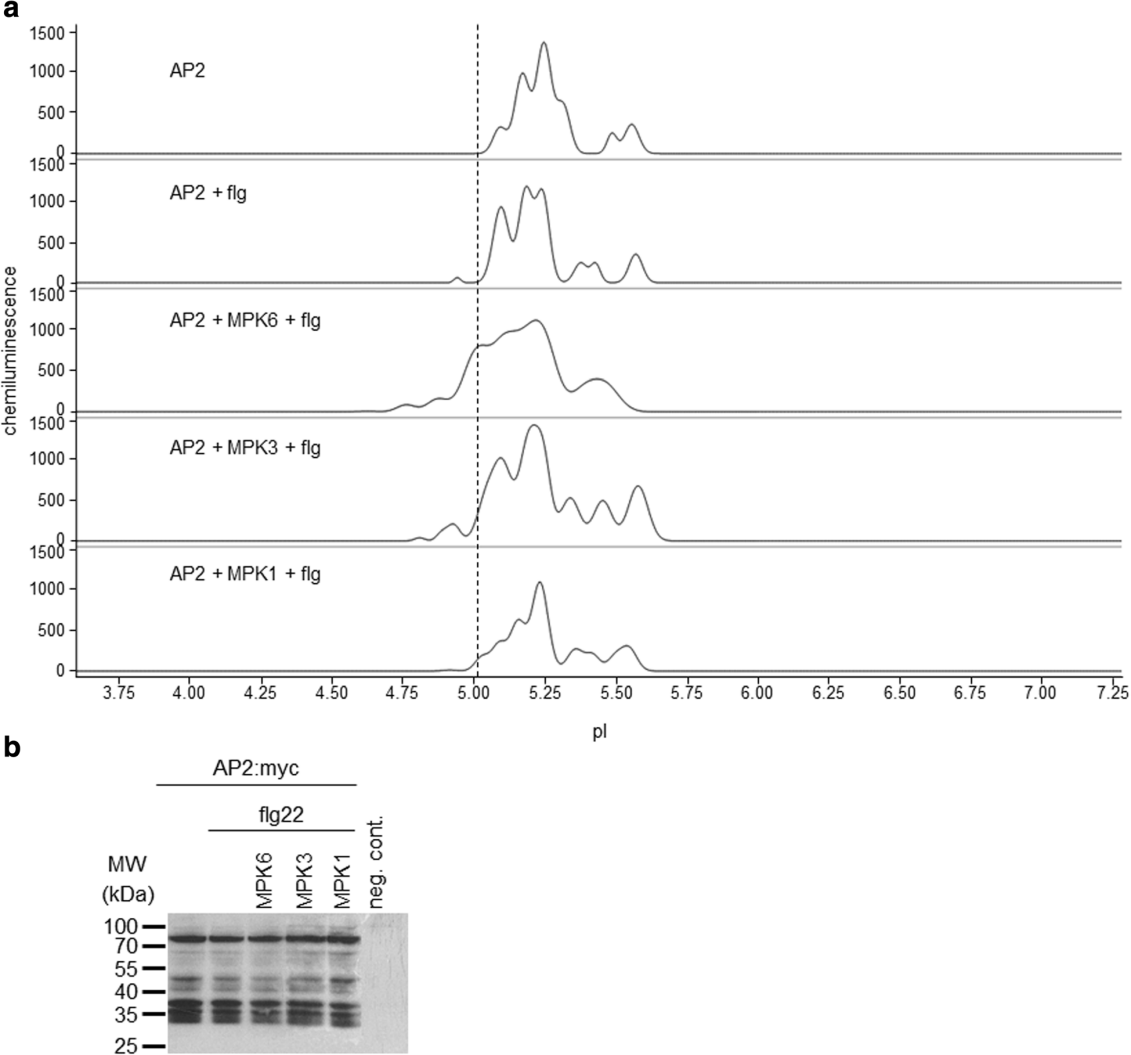
AP2 is an MPK6 substrate in vivo. **a** Effect of MAPK co-expression and flg treatment on C-terminal myc-tagged AP2 isoform distributions in cIEF-immunoassay. Expressed proteins and treatments are indicated for each sample. Dashed line indicates the position of a major acidic isoform specifically appearing in the presence of activated MPK6. **b** Conventional SDS-PAGE immunoblot of transiently expressed AP2:myc variants in the samples corresponding to panel **a**. Negative control (neg. cont.) denotes a protoplast sample not transfected with the myc epitope

The homeotic gene AP2 is a key floral regulator and according to the ABC model of flower development AP2 is a type-A transcription factor [28, 29]. Although, similarly to WUS, AP2 function is extensively characterised by genetic means, post-translational modification of AP2 has not been reported yet. Nonetheless, AP2 contains a remarkably high number of putative kinase interaction motifs and phosphorylation sites, (Additional file 9: Table S4). Accordingly, the parallel existence of several AP2 isoforms in untreated cells was detected (Fig. 5a, Table 3). The complex isoform distribution could be significantly reduced by phosphatase treatment of the protein extracts (Additional file 2: Fig. S1).

**Table 3:** Peak data for AP2 phosphorylation

pI	Area %: AP2 +				
	-	flg	MPK6 + flg	MPK3 + flg	MPK1+ flg
4.76	0	0	2.32	0	0
4.81	0	0	0	0.65	0
4.88	0	0	2.27	0.49	0
4.94	0	0	0	3.6	1.62
5.01	0.27	2.32	21.96	8.4	4.49
5.09	7.56	27.44	6.88	16.65	10.69
5.15	0	0	18.03	0	0
5.17	26.23	0	0	0	18.92
5.19	0	16.78	0	23.94	0
5.24	36.49	27.17	29.85	12.42	26.16
5.29	0	0	0	0	14.26
5.32	14.59	0	0	0	0
5.36	0	6.37	0	10.31	6.49
5.43	0	3.81	0	0	7.49
5.46	0	0	18.69	7.94	0
5.49	4.84	0	0	0	0
5.57	10.04	16.1	0	15.58	9.87

Data correspond to the electropherograms shown in Fig. 5a. Peaks 5.15-5.19 may represent the same isoform, not well separated from the neighbouring major peaks. Accumulation of a major isoform of pI ~ 5.0 occurred exclusively in the MPK6 + flg22 samples in three independent biological repeats

We assayed phosphorylation of AP2 by co-expression with activated MAPKs (Fig. 5a, Table 3). Theoretical pI of the AP2:myc fusion is 5.24, which corresponds to the main peak that was consistently detected in all samples. Some acidification can be observed in flg-treated samples (especially isoform of pI ~5.1), indicating phosphorylation by endogenous kinases. When MPK6 was co-expressed, a pronounced acidification of AP2 was observed. In this case a novel major isoform of pI 5.0 appeared. In contrast, the pI 5.0 isoform is not present in samples where AP2 is co-expressed with either the related MPK3 or MPK1. These data imply that AP2 is an MPK6 substrate in vivo. Again, conventional immunoblot is insufficient to resolve alterations in phosphoisoform distribution (Fig. 5b).

## Discussion

Currently phosphorylation of a given protein by a specific kinase is commonly studied by in vitro kinase assays, although due to the use of purified proteins outside of the cellular context this method is prone to false positive and negative results [9, 14]. Furthermore, the use of radioisotopes also means serious safety and environmental hazards. Here we present Kinase-Associated Phosphoisoform Assay, a method which provides an alternative approach to study specific phosphorylation interactions in vivo, and using the novel method we demonstrate phosphorylation of two key plant developmental regulators for the first time. Cloning of substrate-encoding cDNAs into fusion vectors is required for both methods. In case of in vitro kinase assay most commonly GST-fusion proteins are expressed in suitable *E. coli* strains. However, an important difference is that efficient expression of plant proteins in a prokaryotic system can be problematic (e.g. formation of inclusion bodies). Moreover, purification of expressed proteins requires further intense efforts prior to the actual kinase assay reaction, which is then followed by SDS-PAGE separation, with subsequent detection of incorporated radioisotopes by autoradiography. In comparison, with the novel method proteins of interest are expressed in plant cells, and a rapidly obtained crude extract can then be directly applied for cIEF-immunoassay, where separation and detection is carried out within a few hours.

Capillary electrophoresis has brought about ground-breaking advances in biomolecular analysis and when coupled with immunoassay it can overcome many limitations of the cumbersome conventional SDS-PAGE immunoblot method. To the best of our knowledge, this is the first application of cIEF-immunoassay in plant research, and expansion of the kinase experimental toolkit can contribute to narrowing the knowledge gap in cellular signalling between mammalian and plant systems. Transfection-based experiments are commonly used in signalling studies, and protoplast transient expression has been widely used in plant MAPK research [30]*.* Furthermore, instead of relying on specific antibodies for each protein of interest, commercial antibodies for commonly used epitopes are available from several sources, they are specific, reliable and reasonably priced. Capillary electrophoresis is extremely sensitive, it is reportedly capable of detecting proteins from 25-cell samples [15], therefore the amount of transfected cells and plasmid DNA may be significantly reduced to optimally utilise resources.

Protoplasts can be isolated from various types of tissues or from specific mutant plant materials, thus experiments can be specifically designed for specific purposes, e.g. to avoid pairing of proteins that do not exist in the same cell types. The problems associated with overexpression are commonly alleviated by using inducible or cell-type-specific promoters. However, protoplasts offer an outstanding advantage in this respect: transformation occurs synchronously at a given time point, and translated proteins can be detected in a few hours after transformation, which is usually followed by a linear increase for about twenty hours. Therefore, it is possible to fine-tune expression levels in protoplasts by adjusting incubation times [31]. Furthermore, when assaying candidate substrates of a given kinase, protoplasts may be derived from a kinase null mutant background, and the transfected kinase can also be driven by its own promoter.

Importantly, protoplast transfection methods have been developed for a wide range of plant species, some of which are difficult or lengthy to transform [32]. Therefore the novel method can be also applied to directly study signalling in economically important crop species.

We have identified putative substrates, which are exclusively expressed in specific cell types and are thus likely to be missed by most screening experiments, by using an online motif search tool. However it is reasonable to expect that more sophisticated motif search methods will be developed and applied for efficient substrate prediction in the future. For example a machine-learning-based method was developed to identify D-site motifs in human proteins [33]. In *Arabidopsis* novel candidate substrates were predicted for MPK3 and MPK6 by consensus phosphorylation sequences [13]. Such computational methods will certainly benefit from a robust method to rapidly and reliably verify phosphorylation interactions and thereby facilitate iterated models of identification and prediction of kinase substrates.

When addressing biological questions it has to be taken into account that each method has certain strengths and weaknesses. This has to be considered upon experimental design in the context of the prior knowledge, hypotheses and independent lines of evidence. Just as all commonly used cellular methods of the protein interaction toolkit (e.g. co-immunoprecipitation or fragment complementation), due to its cellular nature, the presented assay also does not fully exclude the possibility of indirect phosphorylation interactions. Nevertheless, as the method provides protoplast samples expressing differentially tagged proteins, it is also feasible to use aliquots for parallel interaction assays, which are commonly carried out in protoplasts in kinase-substrate studies. Besides other lines of evidence, if specific interaction sequences are known, direct interaction can be inferred from mutating them.

Scaling up Kinase-Associated Phosphoisoform Assay seems feasible using high-throughput protoplast transformation [34] and publicly available Gateway-based cDNA collections [35]. Besides validating screening results or computational predictions, medium-throughput application of the method could in principle be also used for screening expression libraries.

At present meristem organisation and organ formation are understood in great detail due to decades of intense genetic research. Use of mutant lines led to the identification and characterisation of various master regulator transcription factors. In vivo phosphorylation of two well-characterised regulators, as shown here, suggests that their cellular functions are dynamically modulated, and that post-translational modifications have to be considered to gain accurate functional understanding, for example by using phosphorylation mutant gene versions in transgenic studies.

## Conclusions

The presented method expands the plant kinase experimental toolkit and complements technical advances in unbiased screening and in silico prediction methods. It provides a cutting-edge analytical approach to assay specific kinase-substrate interactions in vivo. Moreover, this method is non-radioactive and also markedly hassle-free in comparison to the in vitro assay. Using a known substrate and various control setups we have demonstrated that the proposed principle to assay differential phosphoisoform distributions is feasible to identify in vivo protein phosphorylation events. The presented experimental approach can be further adjusted and improved for specific purposes, for example by using various promoters, sources of protoplasts or capillaries of other characteristics (e.g. pH resolution). Therefore this strategy can facilitate future progress in unravelling kinase targets in the various regulatory pathways, which comprise the complex adaptation mechanisms of sessile plants.

## Methods

### Generation of expression constructs

Open reading frames were amplified either from newly synthesised cDNA or from cDNA clones obtained from TAIR. Total RNA was isolated from *Arabidopsis* seedlings using RNeasy Plant Mini kit (Qiagen), then cDNA synthesis was carried out by RevertAid First Strand cDNA Synthesis Kit (Thermo Scientific). PCR products were cloned into pGEM-T Easy vector (Promega). Following sequence verification, ORFs were subcloned into a pRT100 [36] derivative using PCR-introduced 5′ NcoI and 3′ NotI restriction sites to generate in-frame C-terminal triple-myc epitope or GFP fusion constructs. The ACS6 C-terminal domain corresponds to amino acid positions 435 to 495 [20] and was C-terminally fused as a 5′ NcoI - 3′ NotI fragment to GFP in pGreenII-0029 vector, driven by a double 35S promoter.

Site-directed mutagenesis reactions were performed using QuikChange Lightning Site-Directed Mutagenesis Kit (Agilent Technologies). Kinase inactive MPK3 was generated by disabling the ATP-binding site as described [37].

For in vitro transcription/translation WUS CDS was subcloned into pEU3-NII-GLICNot vector by ligation independent cloning [38].

Oligos used in this work are presented in Additional file 10: Table S5.

### Protoplast preparation and transfection

The suspension cell culture used in this work was originally initiated from *Arabidopsis thaliana* Col-0 (ecotype Columbia-0) roots in the laboratory of C. Koncz (Max Planck Institute for Plant Breeding Research, Cologne, Germany) [39]. Protoplasts were prepared and transiently transformed as described [40]. Cell culture was initiated from *Arabidopsis* Col-0 roots, and maintained in 4.414 g/l MS + B5 vitamins (Duchefa), 30 g/l sucrose, and 1 mg/l 2,4-D, pH 5.7. Cell cultures were weekly diluted 1:5. Three-day-old cells were collected by centrifuging for 5 min at 290 rcf. Cell walls were removed by agitating at 28 °C in B5-GM medium (3.164 g/l B5 powder (Duchefa), 0.34 M glucose and 0.34 M mannitol, pH 5.5) supplemented with 0.25 % cellulase (Yakult) and 0.05 % macerozyme (Yakult), until cells became spherical (about four hours). Protoplasts were washed once with B5-GM and resuspended in 5 ml B5-SM medium (3.164 g/l B5 powder (Duchefa) and 0.28 M sucrose, pH 5.5). Protoplasts were then separated by floating following centrifugation for 7 min at 130 rcf. After cell counting, protoplast concentration was adjusted to 10^7^ / ml.

Five μg of each plasmid and 5 × 10^5^ protoplasts (in 50 μl) were used for each transformation reaction. The protoplast-DNA mixture was treated with 150 μl PEG solution (25 % PEG 6000, 0.45 M mannitol and 0.1 M Ca(NO_3_)_2_) for 15 min. PEG was washed by 1 ml of 0.275 M Ca(NO_3_)_2_, and protoplasts were resuspended in 0.5 ml of B5-GM. To reduce variations between independent transformation events, three transformation reactions were carried out for each sample, pooled and separated into 5 × 10^5^ and 10^6^ cells for cIEF-immunoassay and immunoblot, respectively. MAPK activation was triggered by treatment of rested protoplasts with 1 μM flg22 peptide (custom synthesised by BioBasic) for 30 min. MG-115 treatments were carried out by addition of MG-115 (Calbiochem) at 100 μM final concentration to the media for 10 min prior to flash freezing the cells.

### cIEF-immunoassay

cIEF-immunoassays were carried out on a NanoPro100 instrument (ProteinSimple). Reagents and consumables were supplied by ProteinSimple. All pipetting steps were carried out at 4 °C in a refrigerator box.

Pelleted, flash-frozen protoplasts were lysed in 100 μl final volume (94 μl Bicine/CHAPS buffer, 4 μl Aqueous Inhibitor mix, 2 μl DMSO Inhibitor mix), by vortexing for 10 s at 4 °C. Lysates were centrifuged at 14,000 rcf for 40 min at 4 °C. Protein concentration of the supernatant was determined by absorbance at 280 nm measured on a NanoDrop spectrophotometer (Thermo Scientific) and set to 0.1 mg/ml final concentration.

146.7 μl Premix G2 pH 3–10 or 5–8 separation gradient was mixed with 3.3 μl pI Standard Ladder by thorough vortexing. 4 μl of cell lysate (0.1 mg/ml) was added to 12 μl of the separation gradient – pI standard mixture per sample, mixed, and 4 μl of each sample was loaded into row ‘A’ of a NanoPro plate. 10 μl of primary antibodies diluted 1:50 in Antibody Diluent solution were loaded into row ‘B’. 10 μl horseradish peroxidase (HRP) conjugated secondary antibody diluted 1:100 in Antibody Diluent solution were loaded into row ‘C’. In case the primary antibody was directly HRP-conjugated, 10 μl Antibody Diluent was loaded into row ‘C’. The plate was then centrifuged at 2,500 rcf for 5 min at 4 °C. Row ‘D’ was loaded with 500 μl of 1:1 mixture of Luminol – Peroxide solutions. Row ‘E’ was loaded with 1800 μl Wash Buffer. Prior to inserting the plate into the sample plate holder and loading the capillary cartridge the separation troughs were loaded with 900 μl electrolyte solution.

Samples were separated and detected according to the following protocol. All steps were programmed and performed automatically in the NanoPro system. Focusing was carried out at 15,000 μW for 30 min. Focused proteins were immobilised within the capillaries by UV illumination for 100 s at factory settings. Incubation time for primary and secondary antibodies was 2 h and 1 h, respectively. Prior to each incubation step the capillaries were washed twice for 150 s. Chemiluminescent detection was carried out for 60, 120, 240, 480 and 600 s. Data were analysed by Compass software (ProteinSimple). All experiments were carried out at least three times, with consistent results.

Lambda protein phosphatase (New England Biolabs) treatments were carried out by supplementing the protein extracts (0.8 μg) in Bicine/CHAPS buffer including Protease Inhibitor Cocktail for plant cell and tissue extracts (Sigma) with NEBuffer for PMP, 1 mM MnCl_2_ and 200 unit phosphatase (New England Biolabs) to 10 μl final volume then incubating at 30 °C for 1 h. The reaction was stopped by heat inactivation of the enzyme at 65 °C. Electropherograms were normalised to the original Bicine/CHAPS buffer system.

Antibodies used in this work are presented in Additional file 8: Table S3.

### Immunoblotting

Protein extracts from protoplasts were prepared in Lacus buffer as described [41]. Equal protein amounts were separated by SDS-PAGE, transferred to polyvinylidene difluoride membranes (Millipore), and probed with antibodies (Table S3).

### In vitro kinase assay

The in vitro mRNA synthesis was carried out using TranscriptAid T7 High Yield Transcription Kit (Thermo Scientific) according to the manufacturer’s instructions. Cell-free translation was carried out by using WEPRO7240H Expression Kit (Cell Free Sciences, Japan). In order to activate His-tagged MPK3 when included in the phosphorylation assay mix, mRNA encoding a constitutively active myc:MKK4 was also added to the translation mixture as described [42].

In vitro-translated His_6_-AtMPK3 proteins were purified by affinity chromatography on TALON Magnetic Beads (Clontech), in vitro-translated GST-WUS and GST-WUS-AA were purified by affinity chromatography on Glutathione Magnetic Beads (Thermo Scientific) [42].

For kinase assays, 300 and 100 ng of in vitro-translated, affinity purified substrate and kinase were used, respectively. As an activity control 10 μg myelin basic protein (MBP) was used as a generic MAPK substrate. The assay was carried out in 20 mM HEPES, pH 7.5, 100 μM ATP, 1 mM DTT, 15 mM MgCl_2_, 5 mM EGTA and 5 μCi [γ-^32^P]ATP with bead-bound GST-WUS or GST-WUS-AA as substrates for 30 min at room temperature, and then stopped by the addition of Laemmli SDS buffer. Samples were fractionated by SDS-PAGE. The gel was fixed, stained with Coomassie Blue, dried and analysed by autoradiography.

## Additional files

Additional file 1: Table S1. Putative kinase and phosphatase interaction (docking) and phosphorylation motifs in the C-terminal domain (pos. 435–495) of ACS6 (1-AMINOCYCLOPROPANE-1-CARBOXYLIC ACID (ACC) SYNTHASE 6). Linear motif search was carried out using the Eukaryotic Linear Motif (ELM) Resource. Putative phosphorylated residues are indicated with red font. Furthermore, the sequence contains a putative D-site MAPK docking motif. This motif contains five spacer residues between the basic cluster and the bulky hydrophobic amino acids, thus falling short of the ELM definition of 2–4 spacers. It is nonetheless commonly accepted that the D-site consensus consists of 1–6 spacer amino acids. (PDF 88 kb) Additional file 2: Figure S1. Effect of lambda phosphatase treatments on protein isoform distribution pattern. Electropherograms of various proteins following lambda phosphatase treatment in cIEF-immunoassay. Expressed proteins are indicated for each sample. (PDF 94 kb) Additional file 3: Table S2. Putative kinase interaction (docking) and phosphorylation motifs in the WUSCHEL protein sequence (292 amino acids). Linear motif search was carried out using ELM. Motifs falling inside SMART/Pfam domains or scoring poorly with the structural filter of ELM are indicated with asterisks. Putative phosphorylated residues are indicated with red font. Furthermore, the sequence contains a putative D-site MAPK docking motif. This motif contains only one spacer residue between the basic cluster and the bulky hydrophobic amino acids, thus falling short of the ELM definition of 2–4 spacers. It is nonetheless commonly accepted that the D-site consensus consists of 1–6 spacer amino acids. (PDF 87 kb) Additional file 4: Figure S2. WUS is an MPK3 substrate in vivo. **a**-**b** Electropherograms of various WUS:GFP fusion proteins and their isoform distributions in cIEF-immunoassay. Expressed proteins and treatments are indicated for each sample. **a** Effect of MAPK co-expression and flg treatment on C-terminal GFP-fused WUS isoform distributions in cIEF-immunoassay. Asterisk indicates an acidic isoform specifically accumulating in the presence of activated MPK3. **b** Amino acid substitutions at the MAPK phosphorylation sites T108, S112 to non-phosphorylatable alanines (WUS-AA:GFP) or phosphomimetic aspartic acids (WUS-DD:GFP) impair WUS phosphorylation by MPK3. Asterisk indicates an acidic isoform specifically accumulating in the presence of activated MPK3. **c-e** Electropherograms of various WUS:myc fusion proteins and their isoform distributions in cIEF-immunoassay. Expressed proteins and treatments are indicated for each sample. **c** Effect of MAPK co-expression and flg treatment on C-terminal myc-tagged WUS isoform distributions in cIEF-immunoassay. **d** Aspartic acid substitutions at the MAPK phosphorylation sites T108, S112 impair WUS:myc phosphorylation by MPK3. **e** Disablement of the MAPK docking site impairs WUS:myc phosphorylation by MPK3. (PDF 165 kb) Additional file 5: Figure S3. Examples of peak area quantification. **a**-**d** Electropherograms of various WUS:myc fusion proteins and their isoform distributions in cIEF-immunoassay. Expressed proteins and treatments are indicated for each sample. Area generated for calculation is visualised in green. Data presented in Table 2. (PDF 149 kb) Additional file 6: Figure S4. Immunodetection of transfected proteins by various antibodies. **a** SDS-PAGE immunoblot of transiently expressed WUS:GFP variants co-expressed with various MPKs. **b** SDS-PAGE immunoblot of transiently expressed WUS:myc co-expressed with various MPKs. Negative control (neg. cont.) is a protoplast sample not transfected with the myc epitope. The right panel shows detection of transiently expressed WUS:myc by a specific anti-WUS antibody. Negative control (neg. cont.) is a protoplast sample not transfected with the WUS:myc construct. **c** SDS-PAGE immunoblot of transiently expressed WUS:myc variants. The proteasome inhibitor MG-115 was used to determine the role of protein degradation in the formation of the lighter bands detected. **d** SDS-PAGE immunoblot of various transiently expressed MPKs used in this study. iMPK3 designates an inactive MPK3 mutant. Negative control (neg. cont.) is a protoplast sample not transfected with the HA epitope. **e** Consistency of WUS:myc detection by cIEF-immunoassay. Transiently expressed WUS:myc was detected by the following antibodies: specific anti-WUS (Agrisera, top panel), HRP-coupled anti-myc (Roche) and anti-myc (Sigma). The antibodies used in this study are presented in detail in Additional File 8 Table S3. (PDF 260 kb) Additional file 7: Figure S5. Conservation of the tandem MAPK phosphorylation sites within WUSCHEL orthologues from various species representing different evolutionary distances from Arabidopsis. Where available, annotated WUSCHEL protein sequences were downloaded and aligned using MUSCLE 3.8. In contrast to the highly conserved homeobox domain, the region downstream of the homeodomain is poorly conserved, no consensus sequence can be defined for this region. The phosphorylation sites determined in this study are at positions T108, S112 in AtWUS. The alignment shown corresponds to AtWUS amino acid positions 97–185, including the last four residues of the homeodomain. Proline-directed Ser/Thr (MAPK) phosphorylation sites are highlighted. Position of the tandem phosphosites is well conserved within the Brassicaceae family, it is somewhat more distant from the homeodomain in other dicot species, and positioned further downstream in monocots. In some species the appearance of third site can be observed. Protein identifiers are as follows: Arabis alpina: KFK40169.1, Camelina sativa: XP_010467591.1, Brassica napus: AFD50636.1, Vitis vinifera: XP_002266323.1, Rhododendron ovatum: AHJ14780.1, Solanum tuberosum: XP_006340731.1, Oryza sativa: CAM32354.1, Zea mays: CAM32346.1. (PDF 82 kb) Additional file 8: Table S3. Antibodies used in this study. (PDF 79 kb) Additional file 9: Table S4. Putative kinase and phosphatase interaction (docking) and phosphorylation motifs in the APETALA 2 (At4g36920.1) protein sequence (432 amino acids). Linear motif search was carried out using ELM. Motifs falling inside SMART/Pfam domains or scoring poorly with the structural filter of ELM are indicated with asterisks. Putative phosphorylated residues are indicated with red font. (PDF 88 kb) Additional file 10: Table S5. Oligonucleotides used in this study. (PDF 80 kb)

## Abbreviations

ACS6: 1-AMINOCYCLOPROPANE-1-CARBOXYLIC ACID (ACC) SYNTHASE 6 (AT4G11280)
AP2: APETALA 2 (AT4G36920)
cIEF: Capillary isoelectric focusing
ELM: Eukaryotic Linear Motif Resource
GFP: Green fluorescent protein
MAPK: Mitogen-activated protein kinase
MPK1: MAP KINASE 1 (AT1G10210)
MPK11: MAP KINASE 11 (AT1G01560)
MPK3: MAP KINASE 3 (AT3G45640)
MPK6: MAP KINASE 6 (AT2G43790)
SDS-PAGE: Sodium dodecyl sulfate polyacrylamide gel electrophoresis
WUS: WUSCHEL 1 (AT2G17950)
