# Supplementary material for: Kinase-Associated Phosphoisoform Assay: a novel candidate-based method to detect specific kinase-substrate phosphorylation interactions in vivo
Source: BMC Plant Biol. 2016 Sep 21;16:204. doi: 10.1186/s12870-016-0894-1 (PMC5031308; doi:10.1186/s12870-016-0894-1)
Supplement: Additional file 1: Table S1. — Putative kinase and phosphatase interaction (docking) and phosphorylation motifs in the C-terminal domain (pos. 435–495) of ACS6 (1-AMINOCYCLOPROPANE-1-CARBOXYLIC ACID (ACC) SYNTHASE 6). Linear motif search was carried out using the Eukaryotic Linear Motif (ELM) Resource. Putative phosphorylated residues are indicated with red font. Furthermore, the sequence contains a putative D-site MAPK docking motif. This motif contains five spacer residues between the basic cluster and the bulky hydrophobic amino acids, thus falling short of the ELM definition of 2–4 spacers. It is nonetheless commonly accepted that the D-site consensus consists of 1–6 spacer amino acids. (PDF 88 kb) [file 12870_2016_894_MOESM1_ESM.pdf]

MOTIF	POSITION	SEQUENCE	ELM ACCESSION
D-site MAPK docking motif	22-32	KKKCWQSNLRL	(similar to ELME000233)
Protein phosphatase-1 (PP1) docking motif RVXF	29-35	NLRLSFS	ELME000137
Pro-directed kinase (MAP kinase) Phosphorylation Site	43-49<br>46-52<br>51-57	GFFSPHS<br>SPHSPVP<br>VPPSPLV	ELME000159
Casein kinase (CK) 1 Phosphorylation site	46-52	SPHSPVP	ELME000063
GSK3 phosphorylation recognition site	30-37	LRLSFSDT	ELME000053
Never in mitosis A (NimA)-related kinase (NEK) 2 phosphorylation site	12-17<br>30-35<br>34-39	MAATTM<br>LRLSFS<br>FSDTRR	ELME000336
protein kinase A (PKA) Phosphorylation site	30-36	LRLSFSD	ELME000062

Additional File 1 Table S1

Putative kinase and phosphatase interaction (docking) and phosphorylation motifs in the C-terminal domain (pos. 435-495) of ACS6 (1-AMINOCYCLOPROPANE-1-CARBOXYLIC ACID (ACC) SYNTHASE 6). Linear motif search was carried out using the Eukaryotic Linear Motif (ELM) Resource. Putative phosphorylated residues are indicated with red font. Furthermore, the sequence contains a putative D-site MAPK docking motif. This motif contains five spacer residues between the basic cluster and the bulky hydrophobic amino acids, thus falling short of the ELM definition of 2-4 spacers. It is nonetheless commonly accepted that the D-site consensus consists of 1-6 spacer amino acids.
