# Supplementary material for: Kinase-Associated Phosphoisoform Assay: a novel candidate-based method to detect specific kinase-substrate phosphorylation interactions in vivo
Source: BMC Plant Biol. 2016 Sep 21;16:204. doi: 10.1186/s12870-016-0894-1 (PMC5031308; doi:10.1186/s12870-016-0894-1)
Supplement: Additional file 2: Figure S1. — Effect of lambda phosphatase treatments on protein isoform distribution pattern. Electropherograms of various proteins following lambda phosphatase treatment in cIEF-immunoassay. Expressed proteins are indicated for each sample. (PDF 94 kb) [file 12870_2016_894_MOESM3_ESM.pdf]

MOTIF	POSITION	SEQUENCE	ELM ACCESSION
MAPK docking motif (D-site)	252-257	RRTLPL	(similar to ELME000233)
Phospho-dependent motif that mediates docking of CDK substrates and regulators to cyclin-CDK-bound Cks1.	106-111	MTTPSS	ELME000358
Cyclin recognition site	229-232<br>253-257<br>50-54*	KPLF<br>RTLPL<br>KELYY	ELME000106
Pro-directed kinase (MAP kinase) Phosphorylation Site	105-111<br>109-115<br>37-43*<br>57-63*	NMTTPSS<br>PSSSPNS<br>TRWTPTT<br>AIRSPTA	ELME000159
CK1 phosphorylation site	112-118<br>172-178	SPNSVMM<br>SSGTECG	ELME000063
CK2 Phosphorylation site	170-176	HASSGTE	ELME000064
GSK3 phosphorylation recognition site	24-31<br>104-111<br>105-112<br>108-115<br>28-35*<br>33-40*<br>101-108*	KSGSGGYT<br>TNMTTPSS<br>NMTTPSSS<br>TPSSSPNS<br>GGYTCRQT<br>RQTSTRWT<br>FNGTNMTT	ELME000053
NEK2 phosphorylation motif	28-33<br>101-106*	GGYTCR<br>FNGTNM	ELME000337
Secondary preference for PKA-type AGC kinase phosphorylation	251-257<br>32-38*	HRRTLPL<br>CRQTSTR	ELME000062
Polo-like kinase phosphorylation site	197-203	QDCSMNY	ELME000147

Additional File 3 Table S2

Putative kinase interaction (docking) and phosphorylation motifs in the WUSCHEL protein sequence (292 amino acids). Linear motif search was carried out using ELM. Motifs falling inside SMART/Pfam domains or scoring poorly with the structural filter of ELM are indicated with asterisks. Putative phosphorylated residues are indicated with red font. Furthermore, the sequence contains a putative D-site MAPK docking motif. This motif contains only one spacer residue between the basic cluster and the bulky hydrophobic amino acids, thus falling short of the ELM definition of 2-4 spacers. It is nonetheless commonly accepted that the D-site consensus consists of 1-6 spacer amino acids.
