# Supplementary material for: Kinase-Associated Phosphoisoform Assay: a novel candidate-based method to detect specific kinase-substrate phosphorylation interactions in vivo
Source: BMC Plant Biol. 2016 Sep 21;16:204. doi: 10.1186/s12870-016-0894-1 (PMC5031308; doi:10.1186/s12870-016-0894-1)
Supplement: Additional file 3: Table S2. — Putative kinase interaction (docking) and phosphorylation motifs in the WUSCHEL protein sequence (292 amino acids). Linear motif search was carried out using ELM. Motifs falling inside SMART/Pfam domains or scoring poorly with the structural filter of ELM are indicated with asterisks. Putative phosphorylated residues are indicated with red font. Furthermore, the sequence contains a putative D-site MAPK docking motif. This motif contains only one spacer residue between the basic cluster and the bulky hydrophobic amino acids, thus falling short of the ELM definition of 2–4 spacers. It is nonetheless commonly accepted that the D-site consensus consists of 1–6 spacer amino acids. (PDF 87 kb) [file 12870_2016_894_MOESM4_ESM.pdf]

a

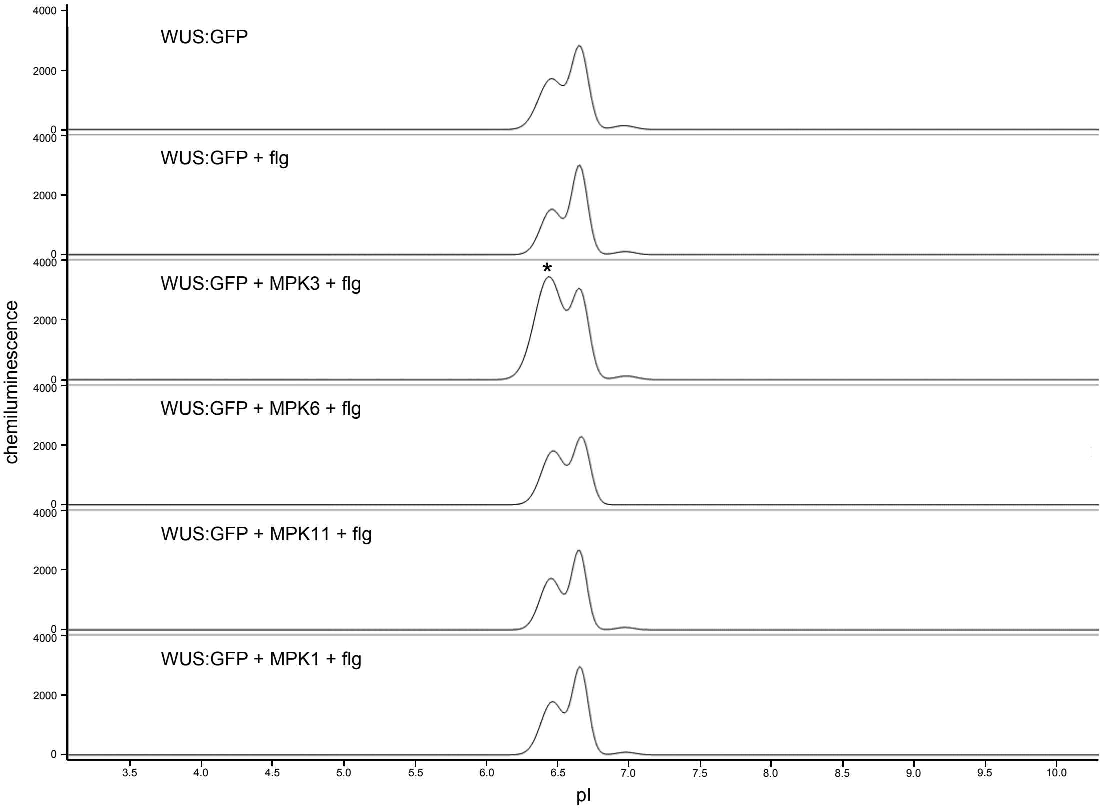

WUS:GFP
WUS:GFP + flg
WUS:GFP + MPK3 + flg
WUS:GFP + MPK6 + flg
WUS:GFP + MPK11 + flg
WUS:GFP + MPK1 + flg
*
chemiluminescence
pI
4000
2000
0
3.5
4.0
4.5
5.0
5.5
6.0
6.5
7.0
7.5
8.0
8.5
9.0
9.5
10.0

b

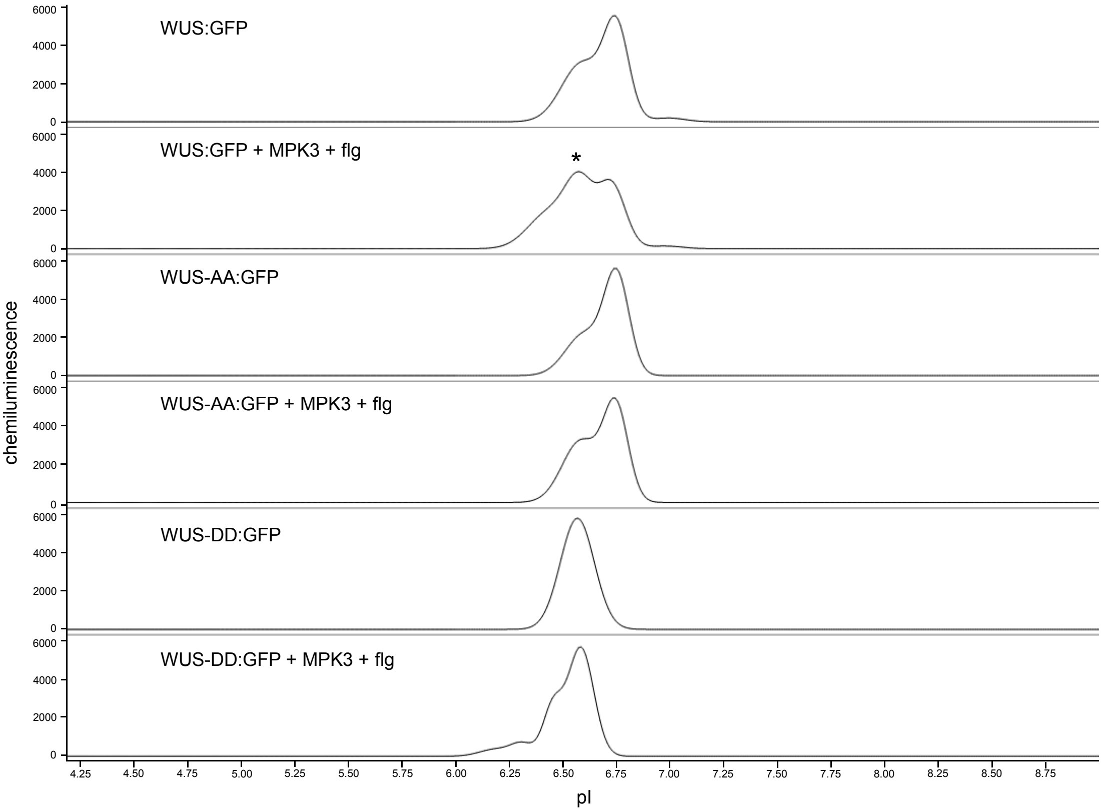

WUS:GFP
WUS:GFP + MPK3 + flg
WUS-AA:GFP
WUS-AA:GFP + MPK3 + flg
WUS-DD:GFP
WUS-DD:GFP + MPK3 + flg
*
chemiluminescence
pI
6000
4000
2000
0
4.25
4.50
4.75
5.00
5.25
5.50
5.75
6.00
6.25
6.50
6.75
7.00
7.25
7.50
7.75
8.00
8.25
8.50
8.75

c

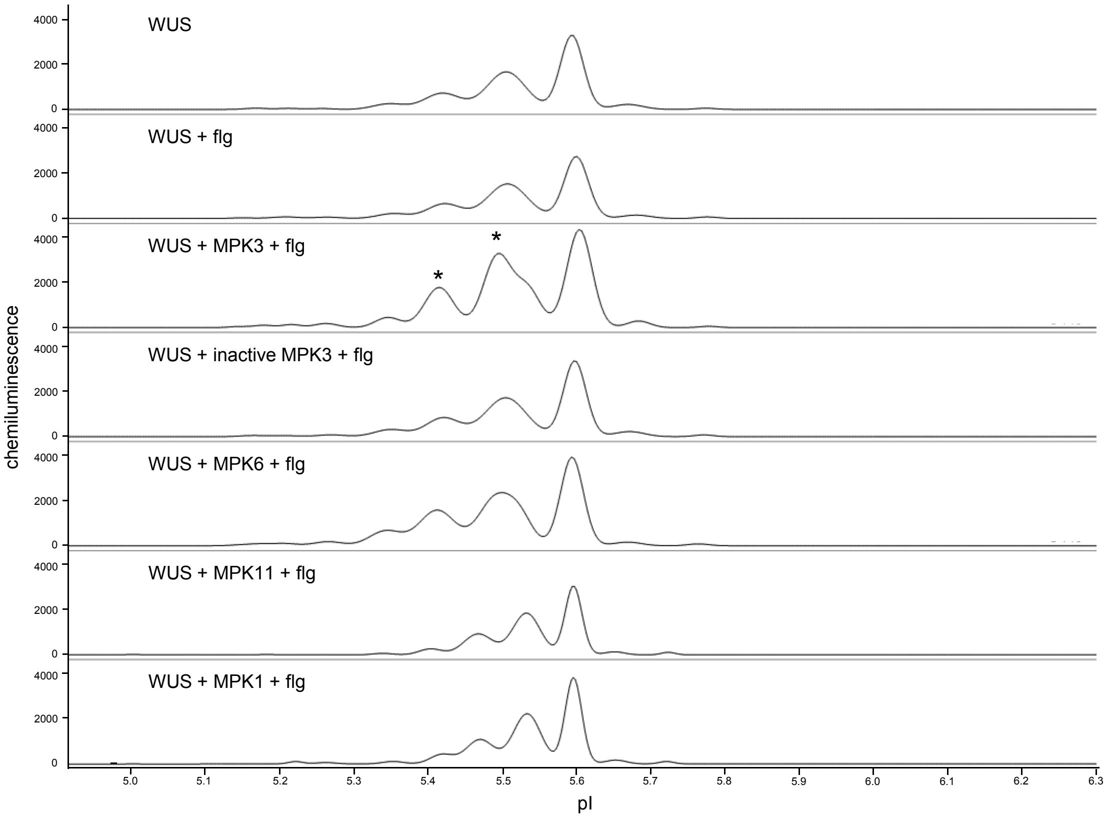
WUS
WUS + flg
WUS + MPK3 + flg
WUS + inactive MPK3 + flg
WUS + MPK6 + flg
WUS + MPK11 + flg
WUS + MPK1 + flg
chemiluminescence
pI

d

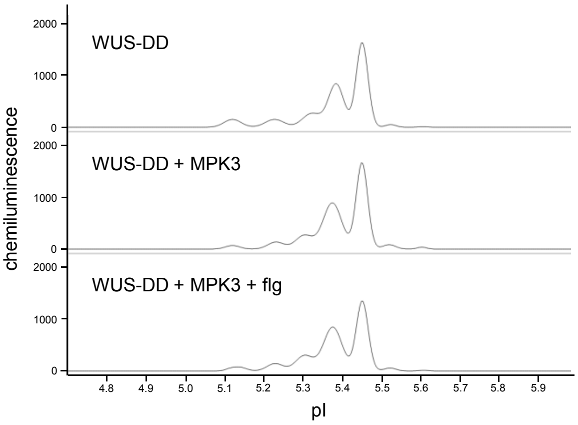
WUS-DD
WUS-DD + MPK3
WUS-DD + MPK3 + flg
chemiluminescence
pI

e

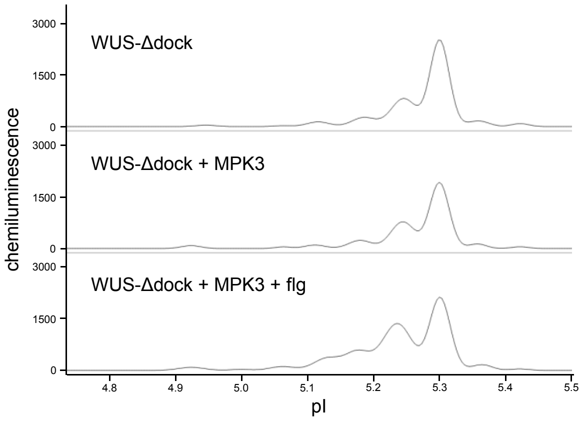
WUS-Δdock
WUS-Δdock + MPK3
WUS-Δdock + MPK3 + flg
chemiluminescence
pI

Additional File 4 Figure S2

WUS is an MPK3 substrate *in vivo*. **a-b** Electropherograms of various WUS:GFP fusion proteins and their isoform distributions in cIEF-immunoassay. Expressed proteins and treatments are indicated for each sample. **a** Effect of MAPK co-expression and flg treatment on C-terminal GFP-fused WUS isoform distributions in cIEF-immunoassay. Asterisk indicates an acidic isoform specifically accumulating in the presence of activated MPK3. **b** Amino acid substitutions at the MAPK phosphorylation sites T108, S112 to non-phosphorylatable alanines (WUS-AA:GFP) or phosphomimetic aspartic acids (WUS-DD:GFP) impair WUS phosphorylation by MPK3. Asterisk indicates an acidic isoform specifically accumulating in the presence of activated MPK3. **c-e** Electropherograms of various WUS:myc fusion proteins and their isoform distributions in cIEF-immunoassay. Expressed proteins and treatments are indicated for each sample. **c** Effect of MAPK co-expression and flg treatment on C-terminal myc-tagged WUS isoform distributions in cIEF-immunoassay. **d** Aspartic acid substitutions at the MAPK phosphorylation sites T108, S112 impair WUS:myc phosphorylation by MPK3. **e** Disablement of the MAPK docking sites impairs WUS:myc phosphorylation by MPK3.
