# Supplementary material for: Kinase-Associated Phosphoisoform Assay: a novel candidate-based method to detect specific kinase-substrate phosphorylation interactions in vivo
Source: BMC Plant Biol. 2016 Sep 21;16:204. doi: 10.1186/s12870-016-0894-1 (PMC5031308; doi:10.1186/s12870-016-0894-1)
Supplement: Additional file 4: Figure S2. — WUS is an MPK3 substrate in vivo. a-b Electropherograms of various WUS:GFP fusion proteins and their isoform distributions in cIEF-immunoassay. Expressed proteins and treatments are indicated for each sample. a Effect of MAPK co-expression and flg treatment on C-terminal GFP-fused WUS isoform distributions in cIEF-immunoassay. Asterisk indicates an acidic isoform specifically accumulating in the presence of activated MPK3. b Amino acid substitutions at the MAPK phosphorylation sites T108, S112 to non-phosphorylatable alanines (WUS-AA:GFP) or phosphomimetic aspartic acids (WUS-DD:GFP) impair WUS phosphorylation by MPK3. Asterisk indicates an acidic isoform specifically accumulating in the presence of activated MPK3. c-e Electropherograms of various WUS:myc fusion proteins and their isoform distributions in cIEF-immunoassay. Expressed proteins and treatments are indicated for each sample. c Effect of MAPK co-expression and flg treatment on C-terminal myc-tagged WUS isoform distributions in cIEF-immunoassay. d Aspartic acid substitutions at the MAPK phosphorylation sites T108, S112 impair WUS:myc phosphorylation by MPK3. e Disablement of the MAPK docking site impairs WUS:myc phosphorylation by MPK3. (PDF 165 kb) [file 12870_2016_894_MOESM5_ESM.pdf]

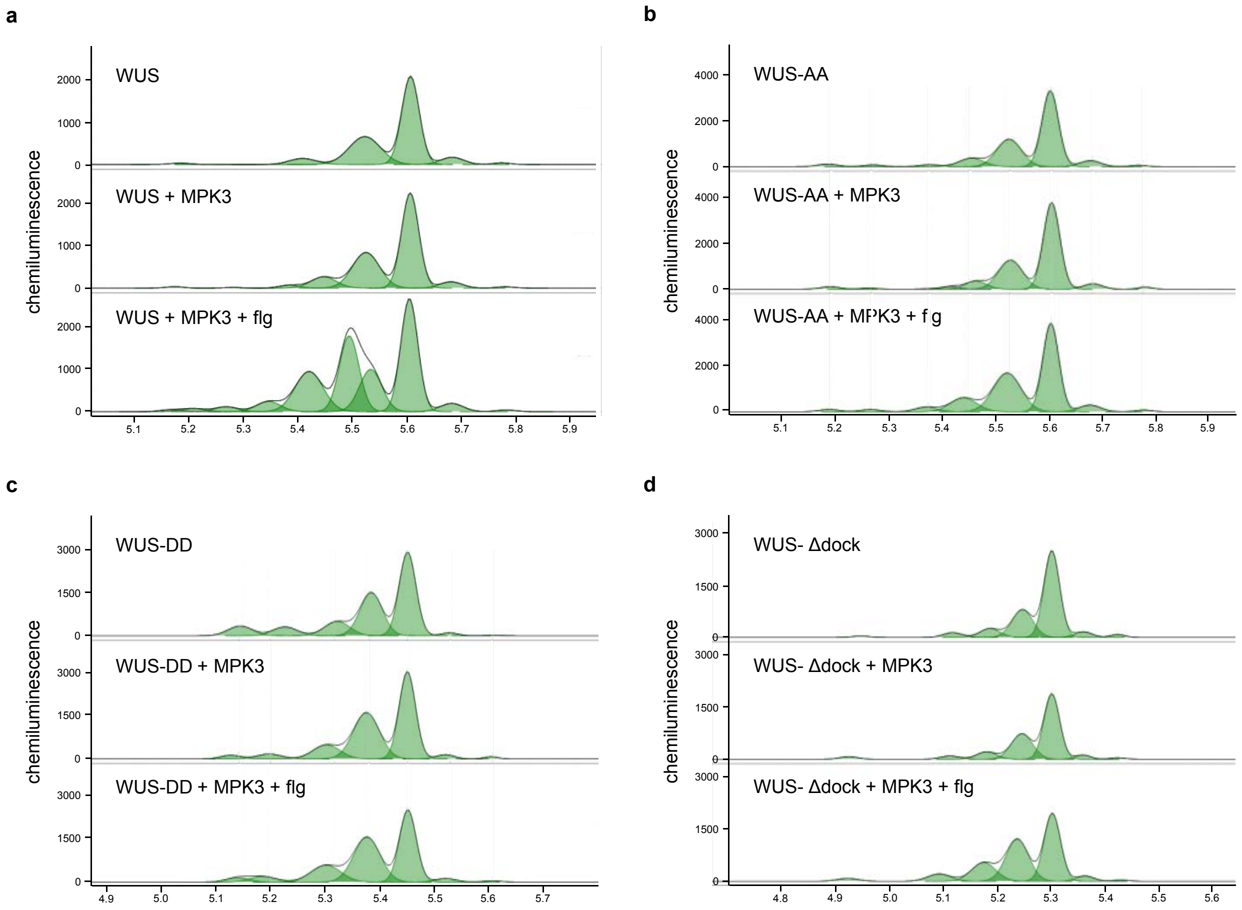

Additional File 5 Figure S3

Examples of peak area quantification. **a-d** Electropherograms of various WUS:myc fusion proteins and their isoform distributions in cIEF-immunoassay. Expressed proteins and treatments are indicated for each sample. Area generated for calculation is visualised in green. Data presented in Table 2.
