# Supplementary material for: Kinase-Associated Phosphoisoform Assay: a novel candidate-based method to detect specific kinase-substrate phosphorylation interactions in vivo
Source: BMC Plant Biol. 2016 Sep 21;16:204. doi: 10.1186/s12870-016-0894-1 (PMC5031308; doi:10.1186/s12870-016-0894-1)
Supplement: Additional file 5: Figure S3. — Examples of peak area quantification. a-d Electropherograms of various WUS:myc fusion proteins and their isoform distributions in cIEF-immunoassay. Expressed proteins and treatments are indicated for each sample. Area generated for calculation is visualised in green. Data presented in Table 2. (PDF 149 kb) [file 12870_2016_894_MOESM6_ESM.pdf]

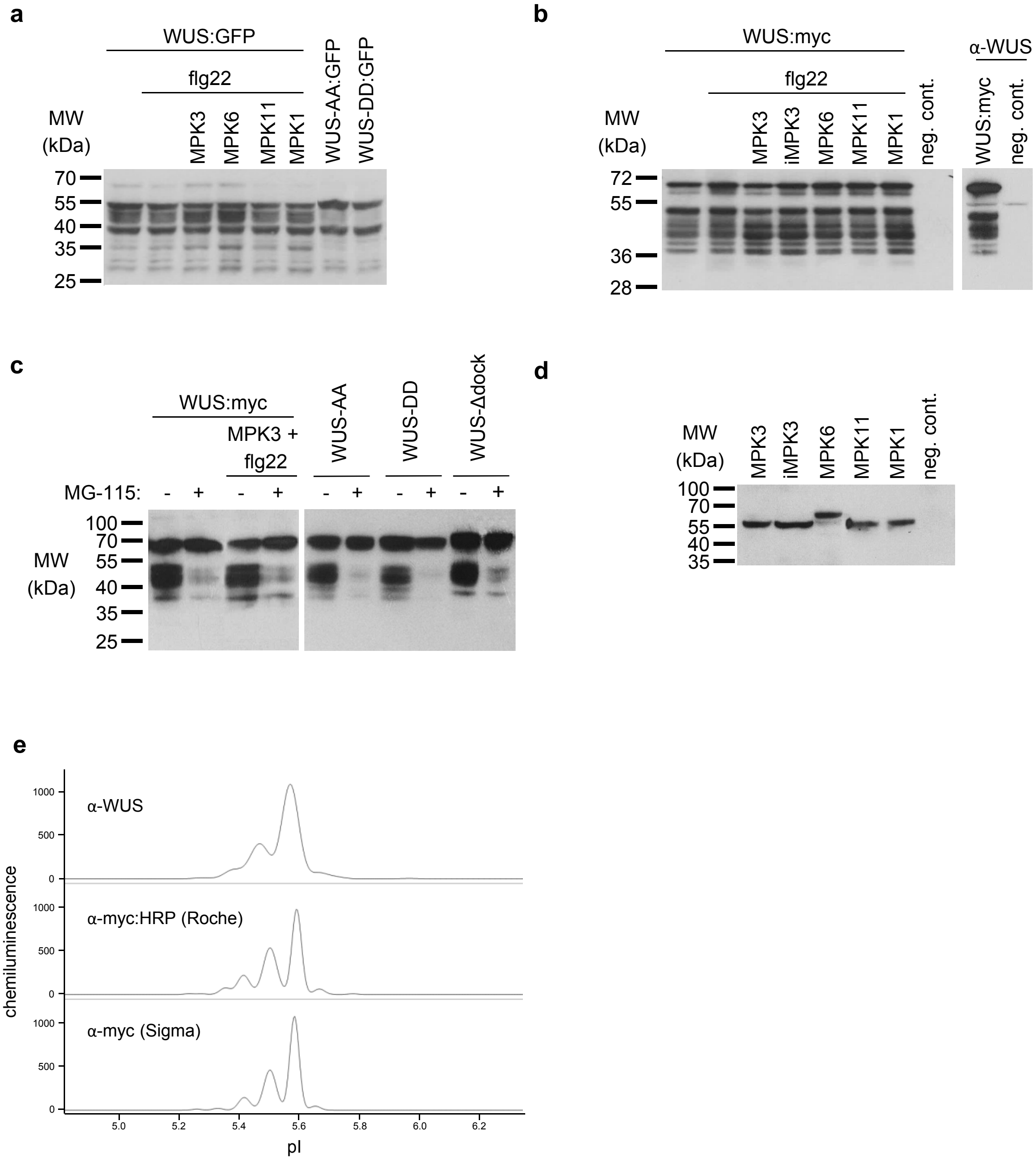
a
WUS:GFP
flg22
MW
(kDa)
MPK3
MPK6
MPK11
MPK1
WUS-AA:GFP
WUS-DD:GFP
70
55
40
35
25
b
WUS:myc
flg22
MW
(kDa)
MPK3
iMPK3
MPK6
MPK11
MPK1
neg. cont.
α-WUS
WUS:myc
neg. cont.
72
55
36
28
c
WUS:myc
MPK3 +
flg22
WUS-AA
WUS-DD
WUS-Δdock
MG-115:
- + - + - + - + - +
100
70
55
40
35
25
MW
(kDa)
d
MW
(kDa)
MPK3
iMPK3
MPK6
MPK11
MPK1
neg. cont.
100
70
55
40
35
e
α-WUS
α-myc:HRP (Roche)
α-myc (Sigma)
chemiluminescence
1000
500
0
5.0
5.2
5.4
5.6
5.8
6.0
6.2
pI

Additional File 6 Figure S4

Immunodetection of transfected proteins by various antibodies. **a** SDS-PAGE immunoblot of transiently expressed WUS:GFP variants co-expressed with various MPKs. **b** SDS-PAGE immunoblot of transiently expressed WUS:myc co-expressed with various MPKs. Negative control (neg. cont.) is a protoplast sample not transfected with the myc epitope. The right panel shows detection of transiently expressed WUS:myc by a specific anti-WUS antibody. Negative control (neg. cont.) is a protoplast sample not transfected with the WUS:myc construct. **c** SDS-PAGE immunoblot of transiently expressed WUS:myc variants. The proteasome inhibitor MG-115 was used to determine the role of protein degradation in the formation of the lighter bands detected. **d** SDS-PAGE immunoblot of various transiently expressed MPKs used in this study. iMPK3 designates an inactive MPK3 mutant. Negative control (neg. cont.) is a protoplast sample not transfected with the HA epitope. **e** Consistency of WUS:myc detection by cIEF-immunoassay. Transiently expressed WUS:myc was detected by the following antibodies: specific anti-WUS (Agrisera, top panel), HRP-coupled anti-myc (Roche) and anti-myc (Sigma).

The antibodies used in this study are presented in detail in Additional File 8 Table S3.
