# Supplementary material for: Kinase-Associated Phosphoisoform Assay: a novel candidate-based method to detect specific kinase-substrate phosphorylation interactions in vivo
Source: BMC Plant Biol. 2016 Sep 21;16:204. doi: 10.1186/s12870-016-0894-1 (PMC5031308; doi:10.1186/s12870-016-0894-1)
Supplement: Additional file 6: Figure S4. — Immunodetection of transfected proteins by various antibodies. a SDS-PAGE immunoblot of transiently expressed WUS:GFP variants co-expressed with various MPKs. b SDS-PAGE immunoblot of transiently expressed WUS:myc co-expressed with various MPKs. Negative control (neg. cont.) is a protoplast sample not transfected with the myc epitope. The right panel shows detection of transiently expressed WUS:myc by a specific anti-WUS antibody. Negative control (neg. cont.) is a protoplast sample not transfected with the WUS:myc construct. c SDS-PAGE immunoblot of transiently expressed WUS:myc variants. The proteasome inhibitor MG-115 was used to determine the role of protein degradation in the formation of the lighter bands detected. d SDS-PAGE immunoblot of various transiently expressed MPKs used in this study. iMPK3 designates an inactive MPK3 mutant. Negative control (neg. cont.) is a protoplast sample not transfected with the HA epitope. e Consistency of WUS:myc detection by cIEF-immunoassay. Transiently expressed WUS:myc was detected by the following antibodies: specific anti-WUS (Agrisera, top panel), HRP-coupled anti-myc (Roche) and anti-myc (Sigma). The antibodies used in this study are presented in detail in Additional File 8 Table S3. (PDF 260 kb) [file 12870_2016_894_MOESM7_ESM.pdf]

```
Solanum tuberosum      QKKRLIAAASATDNNNNNNISPMQMIPHLWRSPDDHH---------------------------KYNNTSTNPGVQCP--------------SASSQGVLAVG
Rhododendron ovatum    QKKRFTTTNTI---ADQIPNI-------LY----NKYPIINTSPAGVP-----------------------TPSSSCC-------------STSSSPAMVAVG
Vitis vinifera         QKKRF---------TTDMPMQRSL-GNAGWRPDDPIH----NKFHTIP-----------------------TPGISSP-------------SSSSSPSVLAVG
Brassica napus         QKKRFNGTTM-TTPTTSSPNSVMM-ANDHYH--HNHHPLLQHHHHGVTMHRPA----SVNIKL-DQENHLLHQNISYPNFHNGNLNHASSGTEYGALNASSNG
Arabis alpina          QKKRFNGTTMTTTPTSSSPNSVMM-ASDRYHHNHHHHPLL-QHHHGVSMQRPA----SLNVKLHDQEHHLLHQNKSYPSFNN--------GSECGAVNA-SNG
A. thaliana            QKKRFNGTNM-TTP-SSSPNSVMMAANDHY------HPLL-HHHHGVPMQRPAN---SVNVKL-NQDHHLYHHNKPYPSFNNGNLNHASSGTECGVVNA-SNG
Camelina sativa        QKKRFNGTTM-TTP-SSSPNSVMM-ANDHY---HQHLPLL-HHHHGVPMQRPAN---SVNVKL-NQEHHLYHQNKSYPSFNNGNLNHANSGTECGAVNA-SNG
Oryza sativa           QKLR----------RRLCISHHLLSCAHYYHHHLAAAAAVVPPPQLLPPLHPSSSSSSCGGGLIDHANSLLSPTSATTP-------------TSAAAAAAAAA
Zea mays               QKMR----------RRLCMSHHLLSCAQYY-------AAAAHHGHAAFLAAPP-----------PYGHQLLSPSTTSP-------------TPAAAAAAAAAA
                       ** *                         :                                                                 .     : .
```

Additional File 7 Figure S5

Conservation of the tandem MAPK phosphorylation sites within WUSCHEL orthologues from various species representing different evolutionary distances from Arabidopsis. Where available, annotated WUSCHEL protein sequences were downloaded and aligned using MUSCLE 3.8. In contrast to the highly conserved homeobox domain, the region downstream of the homeodomain is poorly conserved, no consensus sequence can be defined for this region. The phosphorylation sites determined in this study are at positions T108, S112 in AtWUS. The alignment shown corresponds to AtWUS amino acid positions 97-185, including the last four residues of the homeodomain. Proline-directed Ser/Thr (MAPK) phosphorylation sites are highlighted. Position of the tandem phosphosites is well conserved within the Brassicaceae family, it is somewhat more distant from the homeodomain in other dicot species, and positioned further downstream in monocots. In some species the appearance of third site can be observed. Protein identifiers are as follows: Arabis alpina: KFK40169.1, Camelina sativa: XP_010467591.1, Brassica napus: AFD50636.1, Vitis vinifera: XP_002266323.1, Rhododendron ovatum: AHJ14780.1, Solanum tuberosum: XP_006340731.1, Oryza sativa: CAM32354.1, Zea mays: CAM32346.1.
