# Supplementary material for: Kinase-Associated Phosphoisoform Assay: a novel candidate-based method to detect specific kinase-substrate phosphorylation interactions in vivo
Source: BMC Plant Biol. 2016 Sep 21;16:204. doi: 10.1186/s12870-016-0894-1 (PMC5031308; doi:10.1186/s12870-016-0894-1)
Supplement: Additional file 7: Figure S5. — Conservation of the tandem MAPK phosphorylation sites within WUSCHEL orthologues from various species representing different evolutionary distances from Arabidopsis. Where available, annotated WUSCHEL protein sequences were downloaded and aligned using MUSCLE 3.8. In contrast to the highly conserved homeobox domain, the region downstream of the homeodomain is poorly conserved, no consensus sequence can be defined for this region. The phosphorylation sites determined in this study are at positions T108, S112 in AtWUS. The alignment shown corresponds to AtWUS amino acid positions 97–185, including the last four residues of the homeodomain. Proline-directed Ser/Thr (MAPK) phosphorylation sites are highlighted. Position of the tandem phosphosites is well conserved within the Brassicaceae family, it is somewhat more distant from the homeodomain in other dicot species, and positioned further downstream in monocots. In some species the appearance of third site can be observed. Protein identifiers are as follows: Arabis alpina: KFK40169.1, Camelina sativa: XP_010467591.1, Brassica napus: AFD50636.1, Vitis vinifera: XP_002266323.1, Rhododendron ovatum: AHJ14780.1, Solanum tuberosum: XP_006340731.1, Oryza sativa: CAM32354.1, Zea mays: CAM32346.1. (PDF 82 kb) [file 12870_2016_894_MOESM8_ESM.pdf]

| Antibody | Type | Manufacturer | Cat. no. | Dilution | |
|---|---|---|---|---|---|
| | | | | WB | cIEF-immunoassay |
| anti-GFP | rabbit, polyclonal, primary | GenScript | A-01704 | 1:1000 | 1:50 |
| anti-myc | rabbit, polyclonal, primary | Sigma | C3956 | n.a. | 1:50 |
| anti-WUS | rabbit, polyclonal, primary | Agrisera | AS11 1759 | 1:1000 | 1:50 |
| anti-rabbit-HRP | goat, polyclonal, secondary | GenScript | A00098 | 1:5000 | 1:100 |
| anti-myc-HRP | mouse, monoclonal, HRP-conjugated | Roche | 11814150001 | 1:5000 | 1:50 |
| anti-HA | mouse, monoclonal, primary | Roche | 11583816001 | 1:1000 | n.a. |
| anti-mouse-HRP | rabbit, polyclonal, secondary | Thermo Scientific (Pierce) | 31451 | 1:1000 | n.a. |

**Additional File 8 Table S3**
Antibodies used in this study
