# Supplementary material for: Kinase-Associated Phosphoisoform Assay: a novel candidate-based method to detect specific kinase-substrate phosphorylation interactions in vivo
Source: BMC Plant Biol. 2016 Sep 21;16:204. doi: 10.1186/s12870-016-0894-1 (PMC5031308; doi:10.1186/s12870-016-0894-1)
Supplement: Additional file 8: Table S3. — Antibodies used in this study. (PDF 79 kb) [file 12870_2016_894_MOESM9_ESM.pdf]

MOTIF	POSITION	SEQUENCE	ELM ACCESSION
MAPK docking motif	315-323<br>245-252*	KHKSQDMRL<br>KKYVYLGL	ELME000233
Protein phosphatase-1 (PP1) docking motif	131-138*	QYRGVTFY	ELME000137
Phospho-dependent motif that mediates docking of CDK substrates and regulators to cyclin-CDK-bound Cks1.	296-301	PTTPQD	ELME000358
Pro-directed kinase (MAP kinase) Phosphorylation Site	21-27<br>295-301<br>347-353<br>397-403<br>410-416	CYSSPSK<br>NPTTPQD<br>LNHTPNS<br>SGFSPHH<br>STSTPHQ	ELME000159
Cyclin dependent protein kinase (CDK) Phosphorylation Site	21-27	CYSSPSK	ELME000153
CK1 phosphorylation site	23-29<br>33-39<br>35-41<br>311-317<br>332-338<br>397-403<br>410-416<br>127-133*	SSPSKRV<br>SNSSSSA<br>SSSSAVV<br>SANSKHK<br>SLHSNEV<br>SGFSPHH<br>STSTPHQ<br>SRSSQYR	ELME000063
CK2 Phosphorylation site	8-14<br>199-205<br>252-258*	PHQTQRE<br>TNLTKEE<br>LFDTEVE	ELME000064
GSK3 phosphorylation recognition site	28-35<br>30-37<br>90-97<br>290-297<br>304-311<br>311-318<br>358-365<br>393-400	RVGSFSNS<br>GSFSNSSS<br>FCQSDLAT<br>AESSGNPT<br>LDLSLGNS<br>SANSKHKS<br>PGSSNIGS<br>AAASSGFS	ELME000053
NEK2 phosphorylation site	20-25<br>32-37<br>118-123<br>304-309<br>308-313<br>341-346<br>408-413	FCYSSP<br>FSNSSS<br>LKKSRR<br>LDLSLG<br>LGNSAN<br>LGQTGM<br>FNSTST	ELME000336
NEK2 phosphorylation site	367-372	GGFSLF	ELME000337
phosphoinositide-3-OH-kinase related kinase (PIKK) phosphorylation site	8-14<br>315-321<br>127-133*	PHQTQRE<br>KHKSQDM<br>SRSSQYR	ELME000202
PKA Phosphorylation site	211-217	RRQSTGF	ELME000008

PKA Phosphorylation site	211-217<br>382-388<br>127-133*<br>138-144*<br>218-224*	RRQSTGF<br>GRASTNQ<br>SRSSQYR<br>YRRTGRW<br>PRGSSKY	ELME000062
Polo-like kinase phosphorylation site	304-310<br>279-285*	LDLSLGN<br>FDPSIYD	ELME000147

Additional File 9 Table S4

Putative kinase and phosphatase interaction (docking) and phosphorylation motifs in the APETALA 2 (At4g36920.1) protein sequence (432 amino acids). Linear motif search was carried out using ELM. Motifs falling inside SMART/Pfam domains or scoring poorly with the structural filter of ELM are indicated with asterisks. Putative phosphorylated residues are indicated with red font.
