# Supplementary material for: Kinase-Associated Phosphoisoform Assay: a novel candidate-based method to detect specific kinase-substrate phosphorylation interactions in vivo
Source: BMC Plant Biol. 2016 Sep 21;16:204. doi: 10.1186/s12870-016-0894-1 (PMC5031308; doi:10.1186/s12870-016-0894-1)
Supplement: Additional file 9: Table S4. — Putative kinase and phosphatase interaction (docking) and phosphorylation motifs in the APETALA 2 (At4g36920.1) protein sequence (432 amino acids). Linear motif search was carried out using ELM. Motifs falling inside SMART/Pfam domains or scoring poorly with the structural filter of ELM are indicated with asterisks. Putative phosphorylated residues are indicated with red font. (PDF 88 kb) [file 12870_2016_894_MOESM10_ESM.pdf]

Target	Forward	Reverse
**Generation of expression constructs**		
ACS-C	5' - GGCCATGGTGTTCACTAGCCAACTTGAGGAGG - 3'	5'- CCGCGGCCGCTTAAGTCTGTGCACGGACTAG - 3'
WUS	5' - GGCCATGGAGCCGCCACAGCATCAGCATC - 3'	5'- GCGGCCGCAGTTCAGACGTAGCTCAAGAGAAGCG - 3'
WUS (for subcloning into pEU3-NII-GLICNot)	5' - TACTTCCAATCCAATGCAATGGAGCCGCCACAGCAT - 3'	5' - TTATCCACTTCCAATGTCAGCAGTTCAGACGTAGCTC - 3'
AP2	5'- GGCCATGGGGGATCTAAACGACGCAC - 3'	5'- GCGGCCGCAAGAAGGTCTCATGAGAGG - 3'
**Site-directed mutagenesis**		
ACS-C-AAA	5' - GGCTTCTTCGCGCCTCATGCACCGGTGCCGCCTGCTCCGCTAGTCCG - 3'	5' - CGGACTAGCGGAGCAGGCGGCACCGGTGCATGAGGCGCGAAGAAGCC - 3'
WUS-AA	5' - CAAACATGACCGCACCATCTAGCGCTCCCAACTCGGTTATGATGGC - 3'	5' - GCCATCATAACCGAGTTGGGAGCGCTAGATGGTGCGGTCATGTTTG - 3'
WUS-DD	5' - CAAACATGACCGATCCATCTGTCGACCCGAACTCGGTTATGATGGC - 3'	5' - GCCATCATAACCGAGTTCGGGTCGACAGATGGATCGGTCATGTTTG - 3'
WUS-Δdock	5' - GGCGATGCTTATCTGGAACATGAAGAAACGCTTCCTGAATTCCCTATGCACGG - 3'	5' - CCGTGCATAGGGAATTCAGGAAGCGTTTCTTCATGTTCCAGATAAGCATCGCC - 3'
inactive MPK3	5' - GACGAACGAGCTCGTAGCGATGAGGAAGATAGCTAATG - 3'	5'- CATTAGCTATCTTCCTCATCGCTACGAGCTCGTTCGTC - 3'

Additional File 10 Table S5

Oligonucleotides used in this study
