# Supplementary material for: Kinase-Associated Phosphoisoform Assay: a novel candidate-based method to detect specific kinase-substrate phosphorylation interactions in vivo
Source: BMC Plant Biol. 2016 Sep 21;16:204. doi: 10.1186/s12870-016-0894-1 (PMC5031308; doi:10.1186/s12870-016-0894-1)
Supplement: Additional file 10: Table S5. — Oligonucleotides used in this study. (PDF 80 kb) [file 12870_2016_894_MOESM2_ESM.pdf]

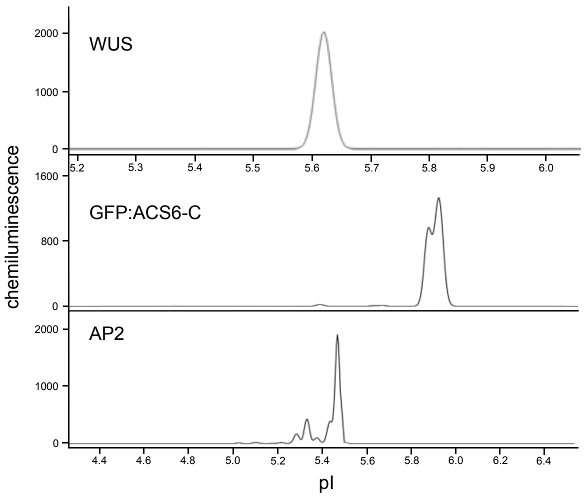

Additional File 2 Figure S1:

Effect of lambda phosphatase treatments on protein isoform distribution pattern. Electropherograms of various proteins following lambda phosphatase treatment in cIEF-immunoassay. Expressed proteins are indicated for each sample.
